# Structures of the sulfite detoxifying F_420_-dependent enzyme from *Methanococcales*

**DOI:** 10.1038/s41589-022-01232-y

**Published:** 2023-01-19

**Authors:** Marion Jespersen, Antonio J. Pierik, Tristan Wagner

**Affiliations:** 1 Max Planck Institute for Marine Microbiology, Bremen, Germany; 2 Biochemistry, Faculty of Chemistry, University of Kaiserslautern-Landau, Kaiserslautern, Germany

**Keywords:** Enzymes, X-ray crystallography, Microbiology, Biocatalysis

## Abstract

Methanogenic archaea are main actors in the carbon cycle but are sensitive to reactive sulfite. Some methanogens use a sulfite detoxification system that combines an F_420_H_2_-oxidase with a sulfite reductase, both of which are proposed precursors of modern enzymes. Here, we present snapshots of this coupled system, named coenzyme F_420_-dependent sulfite reductase (Group I Fsr), obtained from two marine methanogens. Fsr organizes as a homotetramer, harboring an intertwined six-[4Fe–4S] cluster relay characterized by spectroscopy. The wire, spanning 5.4 nm, electronically connects the flavin to the siroheme center. Despite a structural architecture similar to dissimilatory sulfite reductases, Fsr shows a siroheme coordination and a reaction mechanism identical to assimilatory sulfite reductases. Accordingly, the reaction of Fsr is unidirectional, reducing sulfite or nitrite with F_420_H_2_. Our results provide structural insights into this unique fusion, in which a primitive sulfite reductase turns a poison into an elementary block of life.

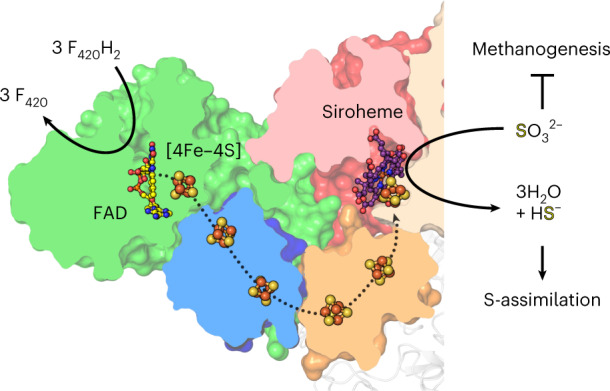

## Abstract (web summary)

The F_420_-dependent sulfite reductase protects some methanogenic archaea by converting toxic sulfite. Structural analysis reveals how the two active centers are electro-connected and provides a plausible picture of a primitive sulfite reductase.

## Main

When cold seawater permeates through sediments or enters hydrothermal vent walls, a partial oxidation of sulfide (HS^−^, S^2−^) results in the formation of (bi)sulfite (HSO_3_^−^), SO_3_^2−^, a highly reactive intermediate of the sulfur cycle^1^. Methanogenic archaea are extremely sensitive to this strong nucleophile, which results in the collapse of methanogenesis, their central energy metabolism^2^. Despite its toxic effects, many hydrogenotrophic methanogens thrive in environments where they are exposed to fluctuating SO_3_^2−^ concentrations, especially methanogens living in proximity to hydrothermal vents or in geothermally heated sea sediments^3–6^.

When exposed to SO_3_^2−^, the hyperthermophile *Methanocaldococcus jannaschii*^3^ expresses high amounts of the Group I coenzyme F_420_-dependent sulfite reductase (referred to as *Mj*Fsr), which confers not only protection, but also the ability to grow on SO_3_^2−^ as sole sulfur source (for example, in the absence of S^2−^)^5,7^. Because of this trait, the *fsr* gene has been used as a genetic marker^7,8^.

Fsr is composed of an N-terminal half belonging to the F_420_-reducing hydrogenase β-subunit family (FrhB; Supplementary Fig. 1) and a C-terminal half made of a single sulfite/nitrite reductase repeat^5,9^ (S/NiRR, from here on referred to as sulfite reductase domain). All known sulfite reductases reduce SO_3_^2−^ using a magnetically coupled siroheme‒cysteine‒[4Fe‒4S] center^10^. This metallocofactor is also used by nitrite reductases to reduce nitrite (NO_2_^−^), a side reaction observed in many sulfite reductases^11^.

**Extended Data Fig. 1:**
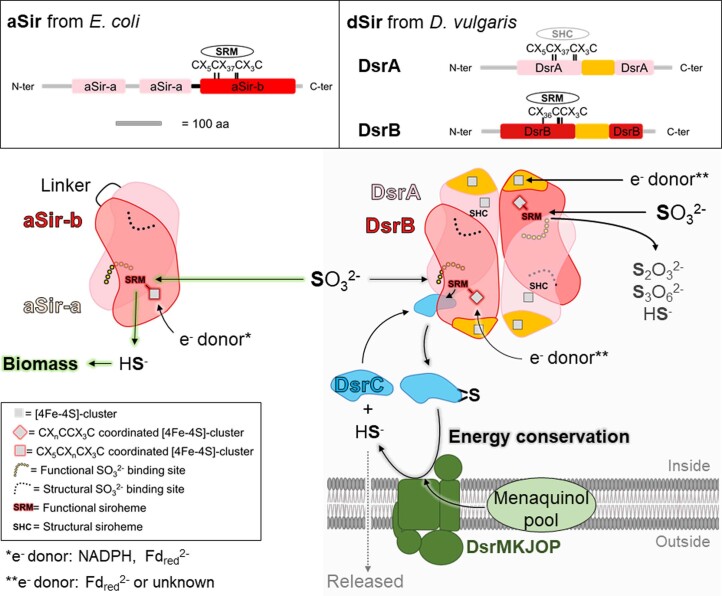
Structural and functional organization of assimilatory (aSir) and dissimilatory (dSir refer here as DsrAB) sulfite reductases. Distinct and conserved domains in aSir as well as dSir are shown in the top panel. The [4Fe-4S]-cluster binding motifs in the proximity of the siroheme or sirohydrochlorin are highlighted. Bottom panel: aSirs (left) are functional monomers that probably evolved through a gene duplication event, where one gene lost its cluster binding motif. The N-terminal half abbreviated as aSir-a (light pink) has a structural function and the C-terminal half abbreviated as aSir-b (red) harbours the active [4Fe-4S]-siroheme. aSirs indirectly use electrons from NADPH (bacteria) or directly via a [2Fe-2S]-cluster containing ferredoxin (plants) to reduce SO_3_^2−^ to HS^−^ in a six-electron reduction reaction^11,35^. The produced sulfide will be used for sulfur assimilation. dSirs (right) are composed of two DsrA (light pink) and two DsrB (red) subunits and receive electrons from reduced ferredoxins (Fd_red_^2−^) or so far unknown donors^15^. In absence of DsrC (cyan), DsrAB turns SO_3_^2−^ to thionates (that is S_2_O_3_^2−^, S_3_O_6_^2−^) and HS^−^. In presence of DsrC, the intermediate sulfur species bound on the siroheme is transferred to DsrC. In the case of *Desulfovibrio* species, the membrane DsrMKJOP complex (green) fully reduces the DsrC-trisulfide (4 electrons transfer) probably by using the menaquinol pool and generates DsrC and HS^−^ via the trisulfide pathway, a key process for energy conservation^15^.

Until now, several groups of sulfite reductases have been identified, which are, depending on their biological function, spectroscopic properties and molecular composition, generally classified into assimilatory or dissimilatory ones, in addition to two biochemically uncharacterized predicted sulfite reductases (Supplementary Fig. 1)^6,11,12^. The only structural data obtained so far are from aSirs (assimilatory) and dSirs (dissimilatory, here, dSirs refer to DsrAB), and therefore this study will use them for comparison. While aSirs are monomeric enzymes that directly reduce SO_3_^2−^ to S^2−^ for assimilation, dissimilatory enzymes are organized by the heterodimers DsrA/DsrB, in which DsrA harbors an inactive catalytic site (referred to as structural; Extended Data Fig. 1)^11–14^. Under physiological conditions, dSirs catalyze the first two-electron reduction step and transfer the sulfur species intermediate to the sulfur-carrier protein DsrC used for energy conservation (Extended Data Fig. 1)^15^. In the absence of DsrC, DsrAB releases some S^2−^, as well as the reaction intermediates trithionate and thiosulfate^15–17^.

Structural and evolutionary studies suggest that aSirs and dSirs originated from a common progenitor^12,14^, a primitive Sir that contained a catalytic siroheme‒[4Fe‒4S] and was operating by itself. The gene encoding this ancestral enzyme was duplicated, and in the dSir case, the duplicated version evolved into DsrB, while DsrA was retained for structural function. In the case of aSir, the original and duplicated genes fused and only one active siroheme‒[4Fe‒4S] was retained. On the basis of sequence and phylogenetic analyses, it has been suggested that *fsr* evolved before the duplication event and therefore represents a primordial sulfite reductase^5,18,19^. Alternatively, *fsr* could have arisen through lateral gene transfer followed by gene fusion events.

Besides its evolutionary importance, the electron-donor module of Fsr, the F_420_H_2_-oxidase, is directly fused to its sulfite reductase domain. This fusion allows the enzyme to perform the entire six-electron reduction of SO_3_^2−^ on its own via an unknown electronic relay, using electrons from reduced F_420_. The coenzyme F_420_ is a deazaflavin derivative present at high cytoplasmic concentrations in methanogens^5,20–22^ and can be reduced by the F_420_-reducing hydrogenase (FrhABG; Supplementary Fig. 1). Due to the difference in the redox potentials of the F_420_/F_420_H_2_ (∆*E*^0^′ = −350 mV) and HSO_3_^−^/HS^−^ (∆*E*^0^′ = −116 mV) couples, the overall reaction is extremely exergonic (∆*G*^0^′ = −135 kJ mol^−1^ per converted SO_3_^2−^) and promotes SO_3_^2−^ detoxification at very high rates^5^. Because of this efficiency and its temperature stability, Fsr is an attractive catalyst for chemists.

Here, we present the X-ray crystal structures of Fsr isolated from two Methanococcales as well as the electron paramagnetic resonance (EPR) spectroscopy characterization of its metallocofactors, providing the first snapshots and molecular insights, to our knowledge, into this prototypical sulfite reductase.

## Results

### Identification of Fsr in *Methanothermococcus thermolithotrophicus*

**Extended Data Fig. 2:**
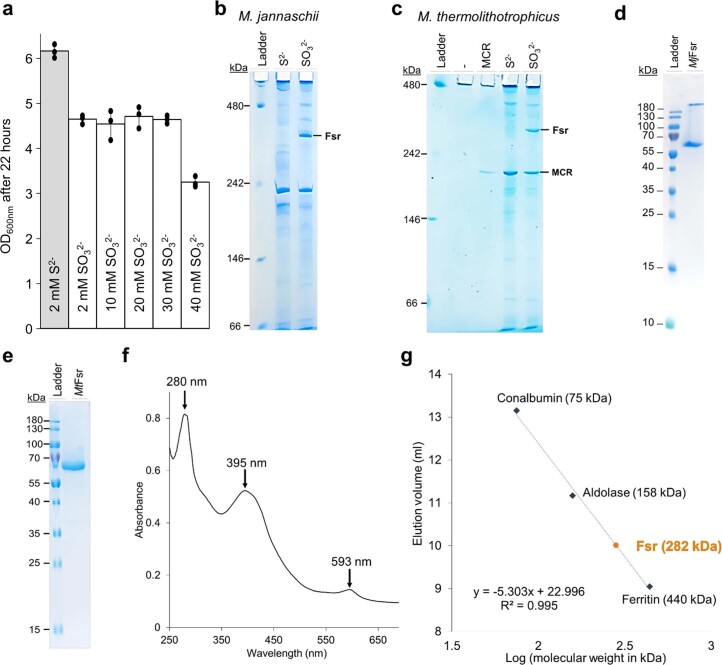
Physiological and biochemical profiles of Fsr from *Methanococcales*. **a**, Final OD_600 nm_ of *M. thermolithotrophicus* grown on sulfide (S^2-^) and different sulfite (SO_3_^2−^) concentrations as a sole sulfur source after 22 hours (mean ± s.d., *n* = 3 biologically independent replicates). **b**, **c**, hrCN-PAGE of cell extracts (12 µg loaded) from *M. jannaschii* (b, *n* = 1 independent experiment) and *M. thermolithotrophicus* (c, *n* = 3 independent experiments), grown on 2 mM Na_2_S or 2 mM Na_2_SO_3_ as a sole sulfur source. Purified MCR from *M. thermolithotrophicus* (1.7 µg loaded) was used as a control for the hrCN-PAGE^54^. **d**, **e**, SDS-PAGE profile of purified *Mj*Fsr (d, *n* = 1 independent experiment) and *Mt*Fsr (e, *n* = 3 independent experiments). **f**,. UV-visible spectrum of 0.33 mg *Mt*Fsr measured anaerobically (100 % N_2_) in 25 mM Tris-HCl, pH 7.6, 150 mM NaCl, 10 % v/v glycerol and 2 mM DTT. *Mt*Fsr displays the typical spectra of [Fe-S]-cluster and siroheme containing enzymes^55^, similar to the UV spectrum of *Mj*Fsr previously determined exhibiting three peaks at 280 nm, 395 nm and 593 nm^5^. **g**, Molecular weight estimation of *Mt*Fsr via size exclusion chromatography (Superdex 200 Increase 10/300 GL from GE Healthcare). Apparent molecular weight of purified *Mt*Fsr (monomeric molecular weight = 69.145 kDa) was estimated to 282 kDa. *Mt*Fsr is therefore apparently organized as a homotetramer (theoretical molecular weight of the protein in the homotetramer: 276.58 kDa). Source data

*Mj*Fsr, previously characterized^5,7,19^, turned out to be less suitable for our structural studies due to crystallization defects (see below). Therefore we took an alternative organism belonging to the same order (*Methanococcales*). *Methanothermococcus thermolithotrophicus* is a fast-growing thermophile isolated from geothermally heated marine sediments that has already demonstrated its advantages for structural biology^23^. It was previously shown that this archaeon can grow on 1 mM SO_3_^2−^ as a sole sulfur source^4^. The participation of Fsr in this process has not yet been investigated and the *fsr* gene appeared to be absent in the 55 contigs of the deposited shotgun genome (assembly number ASM37696v1, Bioproject: PRJNA182394). After adaptation, we confirmed that *M. thermolithotrophicus* could grow on SO_3_^2−^, even at concentrations up to 40 mM (Extended Data Fig. 2a). When cell extracts of both organisms were passed on native PAGE, a distinct band at ≈300 kDa was observed for the cultures grown on SO_3_^2−^ (Extended Data Fig. 2b,c). Based on the band intensity, which is comparable to that of the methyl coenzyme M reductase (MCR, the main catabolic enzyme and one of the highest expressed in the cell), as in *M. jannaschii*^5^, we concluded that Fsr is present in *M. thermolithotrophicus*.

The closed circular genomic sequence of strain DSM 2095 was obtained and contains two entire *fsr* genes, one of which shares 80.4% sequence identity, and a second isoform that shares 75.6% sequence identity with *Mj*Fsr (Supplementary Fig. 2). The purified Fsr in this study has the closest sequence identity (80.4%) to *Mj*Fsr, as confirmed by mass spectrometry.

Fsr from both organisms was purified natively under anaerobic atmosphere and yellow light (Extended Data Fig. 2d,e). SDS–PAGE profiles and sulfite reductase activity assays were used to follow the enzyme during the purification. *Mt*Fsr exhibits the typical absorbance of [4Fe‒4S] clusters and siroheme‒[4Fe‒4S]-containing proteins, as shown for *Mj*Fsr (Extended Data Fig. 2f)^5,24^. Based on the native PAGE and gel filtration profiles, *Mt*Fsr is organized as a homotetramer in solution (Extended Data Fig. 2c,g), similar to *Mj*Fsr^5^.

### The F_420_H_2_-oxidase domain flanks a sulfite reductase core

**Fig. 1:**
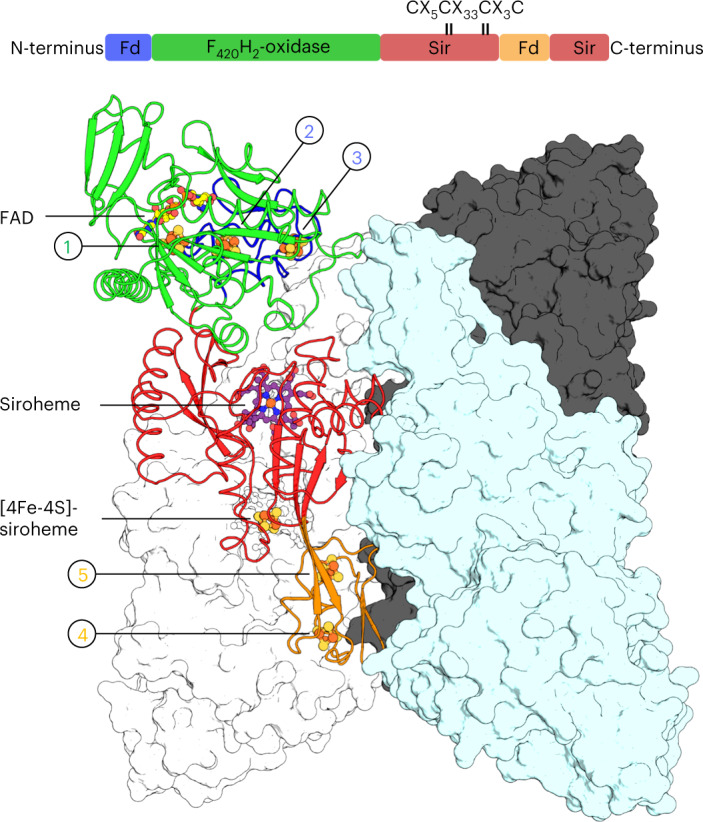
Domain and structural organization of *Mt*Fsr. Visualization of *Mt*Fsr domains (top panel). The [4Fe‒4S] cluster-binding motif in the proximity of the siroheme is highlighted. The main panel shows the tetrameric arrangement of *Mt*Fsr. Three chains are represented in the surface and colored in white, black and cyan. One monomer of *Mt*Fsr is represented as a cartoon and colored according to the top panel. [4Fe‒4S] clusters are numbered on the basis of their position in the electron relay going from the FAD to the siroheme. The siroheme, FAD and the [4Fe‒4S] clusters are represented by balls and sticks. Carbon, nitrogen, oxygen, sulfur and iron atoms are colored as purple (siroheme)/light yellow (FAD), blue, red, yellow and orange, respectively. Fd and Sir stand for ferredoxin domain and sulfite reductase domain, respectively.

**Extended Data Fig. 3:**
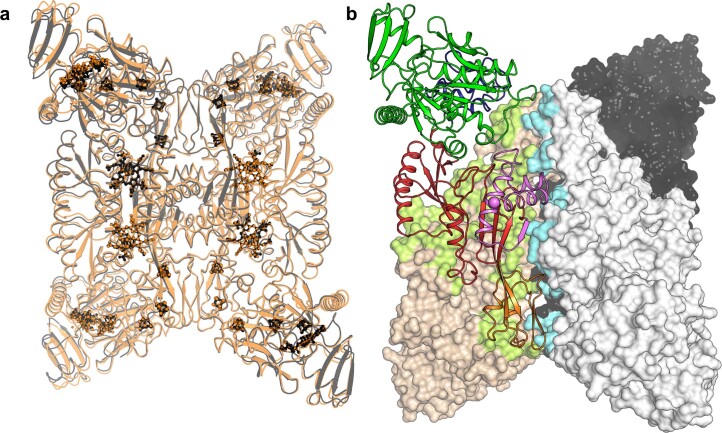
Homotetrameric arrangement of Fsr. **a**, Superposition of *Mt*Fsr (black) with *Mj*Fsr (orange, rmsd of 0.456 Å for 544-Cα aligned). Ligands are shown in balls and sticks and coloured in black and orange for *Mt*Fsr and *Mj*Fsr, respectively. **b**, Surface area involved in the oligomerization in Fsr. Monomers of *Mt*Fsr are shown in surface representation, with one monomer being displayed in cartoon and coloured by its domain composition: the N-terminal ferredoxin domain in dark blue, F_420_H_2_-oxidase in green, the sulfite reductase domain in red and its inserted ferredoxin domain in orange. The C-terminal segment involved in the oligomerization is coloured in light pink with the C-terminus highlighted as a ball. The monomer-monomer contacts are shown as a green surface and contacts to the adjacent dimer are visualized by a cyan surface. The basic monomer-monomer interface of 2,902-Å^2^ for *Mt*Fsr and 2,971-Å^2^ for *Mj*Fsr is established by the sulfite reductase domain and the two additional ferredoxin domains. The C-terminal part of the sulfite reductase domain (562–618 in *Mt*Fsr, 562–620 in *Mj*Fsr), the second ferredoxin domain and the loop 171–189 of the F_420_H_2_-oxidase domain generate the dimer-dimer interface, totalling an area of 3,055-Å^2^ for *Mt*Fsr and 3,037-Å^2^ for *Mj*Fsr. Most of these contacts involve salt bridges. In *Mj*Fsr, the tetrameric structure is supported by two divalent cations, modelled as calcium ions that are each coordinated by a conserved aspartate from the opposite monomers (Asp511 and water molecules)^56^.

A single-wavelength anomalous dispersion experiment was performed to solve the *Mj*Fsr crystal structure. *Mt*Fsr was solved by molecular replacement, using *Mj*Fsr as a template. The crystal structures of both Fsr superpose well (Extended Data Fig. 3a) and were refined to 2.30 Å for *Mj*Fsr and 1.55 Å for *Mt*Fsr (Fig. 1 and Extended Data Table 1). Since *Mj*Fsr has pseudo-merohedral twinning and a lower resolution compared to *Mt*Fsr, the latter was used for the in-depth structural and biochemical analysis.

**Extended Data Fig. 4:**
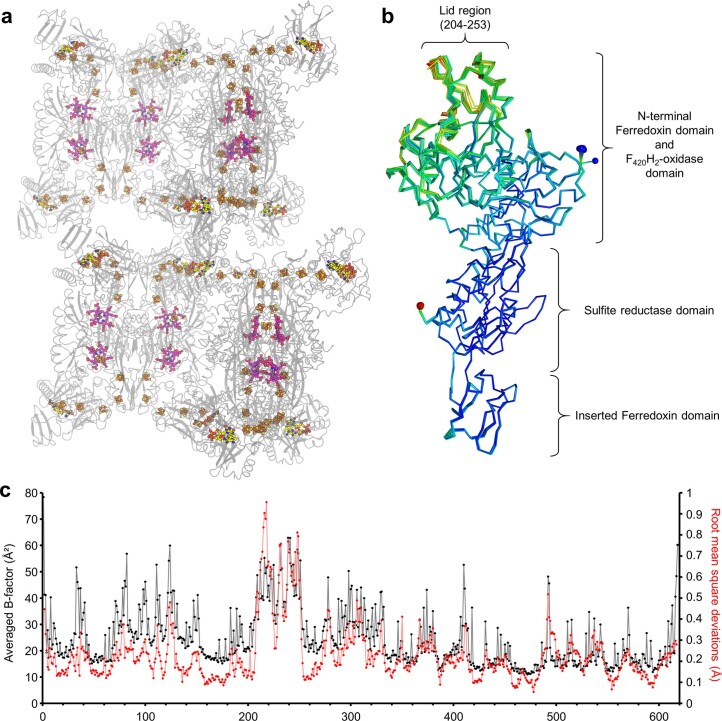
Asymmetric unit content and B-factor profile of *Mt*Fsr. **a**, The four homotetramers contained in the asymmetric unit of *Mt*Fsr are shown in cartoon and the 96 [4Fe-4S]-clusters, the 16 FADs (in yellow) and 16 sirohemes (in pink) are shown in balls and sticks. To our knowledge, *Mt*Fsr contains the highest number of clusters seen in an asymmetric unit so far. **b**, Superposition of all sixteen chains from the asymmetric unit in *Mt*Fsr, with an average rmsd of 0.14 Å for 514-Cα aligned. The N- and C-terminus of each chain are shown by a blue and red sphere, respectively. The models are coloured according to their B-factor values; blue to red indicate low to high B-factors, respectively. **c**, Averaged B-factor values (in black) for each residue from the 16 chains composing the asymmetric unit of *Mt*Fsr. The averaged root mean square deviations (rmsd, in red) of the corresponding Cα is overlaid on the same graph. Averaged rmsd were calculated by the software superpose^57^. Source data

As shown in Fig. 1, Fsr is organized as follows: the N-terminal ferredoxin domain (*Mt*Fsr residues 1–57 containing two [4Fe‒4S] clusters) is linked to the F_420_H_2_-oxidase domain (*Mt*Fsr residues 58–336, harboring the flavin and one [4Fe‒4S] cluster), which is connected to the C-terminal sulfite reductase domain (*Mt*Fsr residues 339–484, 546–618) that binds the siroheme‒[4Fe‒4S] and has an inserted ferredoxin domain (*Mt*Fsr residues 485–545, containing two [4Fe‒4S] clusters). The tetrameric structure of the protein is established by a dimer of two homodimers over a large contact area through the two additional ferredoxin domains and the C-terminal part of the sulfite reductase domain (562–618 in *Mt*Fsr, 562–620 in *Mj*Fsr; Extended Data Fig. 3b). The homotetramer has the overall shape of a butterfly, composed of a sulfite reductase core flanked by the F_420_H_2_-oxidase domain. Notably, the asymmetric unit of *Mt*Fsr contains four tetramers (including 96 [4Fe–4S] clusters), providing insights on its natural flexibility (Extended Data Fig. 4a–c).

**Fig. 2:**
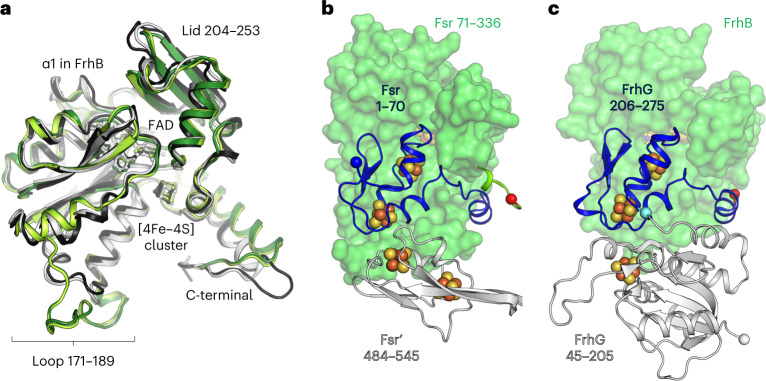
Comparison of the F_420_H_2_-oxidase domain between Fsr and Frh. **a**, Superposition of the F_420_H_2_-oxidase domain in Fsr (*Mj*Fsr in dark green, *Mt*Fsr in light green) with FrhB from *M. barkeri* (black, PDB 6QGR) and FrhB from *M. marburgensis* (white, PDB 4OMF). The extended loops 171–189 in *Mj*Fsr and *Mt*Fsr are highlighted, as well as the lid, which is static in the Frh structures, but more flexible in Fsr (Extended Data Fig. 4b,c). **b**, Representation of *Mt*Fsr F_420_H_2_-oxidase domain (green surface) and its N-terminal ferredoxin domain (blue cartoon residues 1–70). The N terminus of Fsr and C terminus from the F_420_H_2_-oxidase domain are highlighted by blue and red spheres, respectively. The inserted ferredoxin domain, provided by the opposing monomer (Fsr′), is shown in white cartoon representation. **c**, Arrangement of FrhB (green surface) with FrhG (cartoon) from *M. marburgensis* (PDB 4OMF). The N-terminal part (45–205) of FrhG is colored in white and its C-terminal part (206–275), structurally equivalent to the N-terminal ferredoxin domain of Fsr, is colored in blue. The cyan ball highlights the connection between both FrhG parts.

The F_420_H_2_-oxidase domain of Fsr is almost identical between *Mj*Fsr and *Mt*Fsr (root mean square deviation (r.m.s.d.) = 0.33 Å for 277-Cα aligned) and superposes well with FrhB from *Methanothermobacter marburgensis* (PDB 4OMF (ref. 25), with a r.m.s.d. = 0.92 Å for 179-Cα aligned) and *Methanosarcina barkeri* (PDB 6QGR (ref. 26), with a r.m.s.d. = 0.98 Å for 179-Cα aligned; Fig. 2a). The overall fold is perfectly conserved between the F_420_H_2_-oxidase domain of Fsr and FrhB, except for the helix α1 of FrhB, which became a loop in Fsr. The active site of the F_420_H_2_-oxidase domain of Fsr contains a flavin adenine dinucleotide (FAD; Supplementary Fig. 3), which is similarly bound in Fsr and FrhB (Supplementary Fig. 4). No electron density could be found despite cocrystallization with F_420_H_2_ (see Methods). Nevertheless, the reduced F_420_-binding site is presumably located in a positively charged cleft that would complement the charges of the acidic gamma-carboxy groups (Supplementary Fig. 3c)^25,26^.

### A [4Fe‒4S] cluster relay connects both active sites

The distance between the isoalloxazine ring from the FAD to the closest siroheme‒[4Fe‒4S] is approximately 40 Å. Electrons delivered by reduced F_420_ must therefore travel through an electron-transfer relay of metallocofactors. The first part of this relay, located in the N-terminal ferredoxin and F_420_H_2_-oxidase domains, shares high structural homologies with FrhBG. Indeed, FrhG and the N-terminal ferredoxin domain of Fsr are located at the same position of the F_420_-oxidoreductase domain (Fig. 2b,c), resulting in a similar electron relay. This homology suggests a common origin that may have evolved by fusion (for Fsr) or by duplication and fusion (for FrhG).

**Fig. 3:**
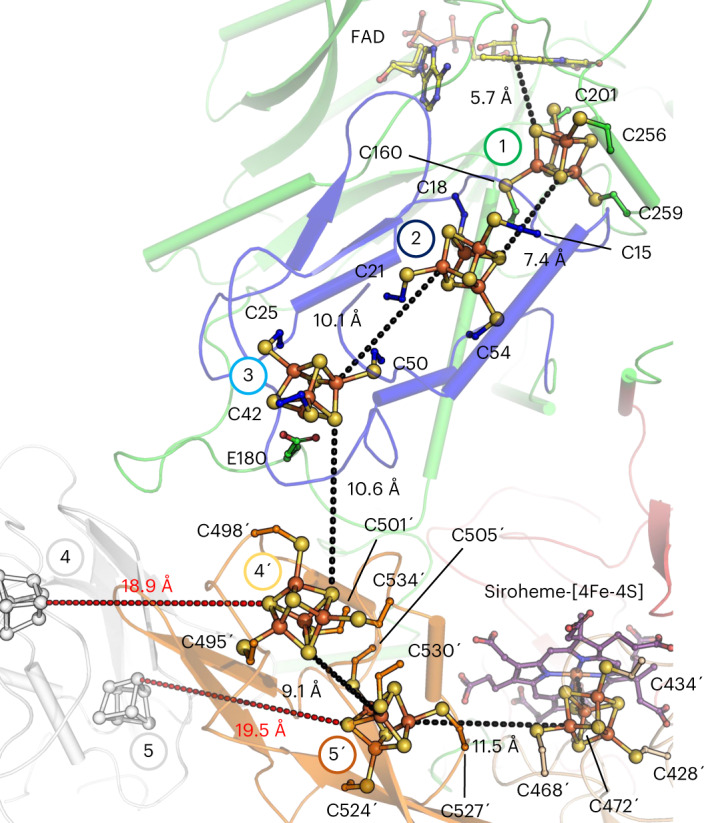
Electron-transfer relay of *Mt*Fsr. *Mt*Fsr, shown as cartoon, has the same color code and numbering of its [4Fe‒4S] clusters (balls and sticks) as in the domain representation in Fig. 1. Edge-to-edge distances connecting the clusters are shown as dashes. The distances to the adjacent [4Fe‒4S] clusters of the opposite dimer are shown in red. The primes correspond to the second monomer forming the dimer. The residues binding the clusters are shown as balls and sticks. Carbon atoms are colored by their domain affiliation. Nitrogen, oxygen, sulfur and iron atoms are colored in blue, red, yellow and orange, respectively. Siroheme and FAD are shown as sticks with purple and yellow carbon atoms, respectively.

As illustrated in Fig. 3, the overall electronic path consists of five [4Fe‒4S] clusters connected by short edge-to-edge distances (<11.5 Å). Dimerization is critical because half of the relay is provided by the second protomer. An intraelectron transfer between both Fsr dimers is unlikely due to the long distance between the nearest clusters (that is, 18.9 and 19.5 Å).

**Extended Data Fig. 5:**
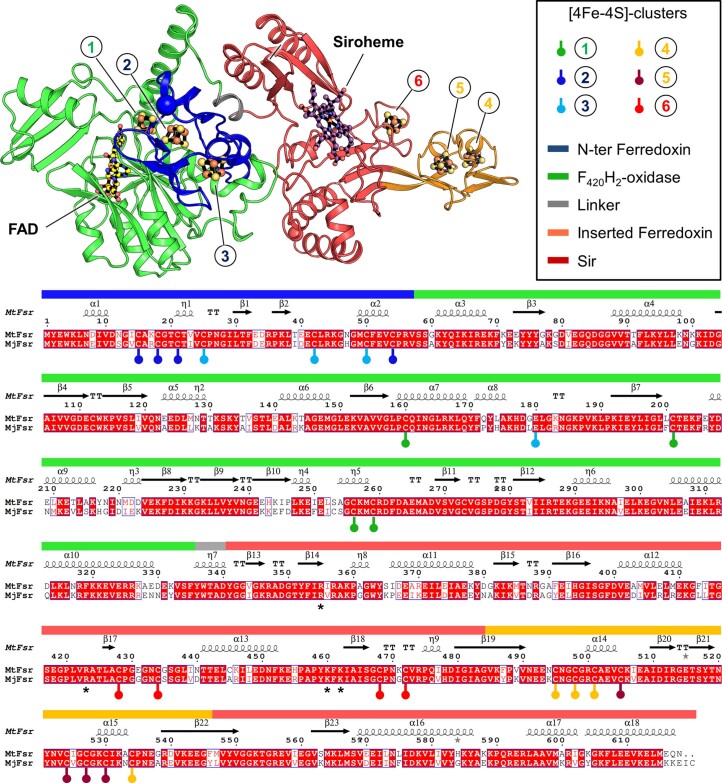
Cluster coordination in Fsr. The top panel shows the monomeric arrangement of *Mt*Fsr (in cartoon) coloured by domains. The siroheme (purple), FAD (yellow) and the [4Fe-4S]-clusters are represented in balls and sticks. Nitrogen, oxygen, sulfur, and iron atoms are coloured respectively, in blue, red, yellow and orange. In the bottom panel, cysteines and the glutamate involved in direct [4Fe-4S]-cluster binding are highlighted, as well as the different domains of Fsr. Sequence alignment was done by Clustal Omega^58^, secondary structure prediction was performed with ESPript 3.0^59^. Cluster 6 is electronically connected to the siroheme. The black stars (*) indicate residues near the siroheme, proposed to bind SO_3_^2−^.

The electrons on the isoalloxazine ring can be transferred directly to the [4Fe‒4S] cluster 1, which is located in the F_420_H_2_-oxidase domain. From there they are passed on to the clusters 2 and 3 in the N-terminal ferredoxin domain. The extended loop 171–189 in Fsr serves as a platform to specifically bind both ferredoxin domains, and the Glu 180 coordinates the [4Fe‒4S] cluster 3 (monodentate, 2.22 Å; Fig. 3, Extended Data Fig. 5 and Supplementary Fig. 5). The electrons continue to flow through the clusters 4′ and 5′ in the inserted ferredoxin domain and finally reach the siroheme‒[4Fe‒4S].

Sequence analyses indicated four [4Fe‒4S] clusters and the one coupled to the siroheme^5^. But both Fsr structures revealed an additional cluster ([4Fe‒4S] cluster 1), which has a noncanonical binding sequence (PCX_40_CX_54_CX_2_C). Strikingly, the four predicted clusters have completely different binding residues compared to primary structural analysis (Extended Data Fig. 5). Each [4Fe‒4S] cluster has a divergent protein environment: cluster 1 is surrounded by basic residues; clusters 2 and 5 have a hydrophobic shell; clusters 4 and 6 are in a more polar environment; and cluster 3 has a glutamate ligand. These differences may reflect the need to establish a ‘redox potential ladder’ to allow a smooth one-way transfer of electrons. To investigate the electron-transfer path, electrochemical experiments followed by EPR spectroscopy were performed.

### Redox properties of the metallocofactors

**Fig. 4:**
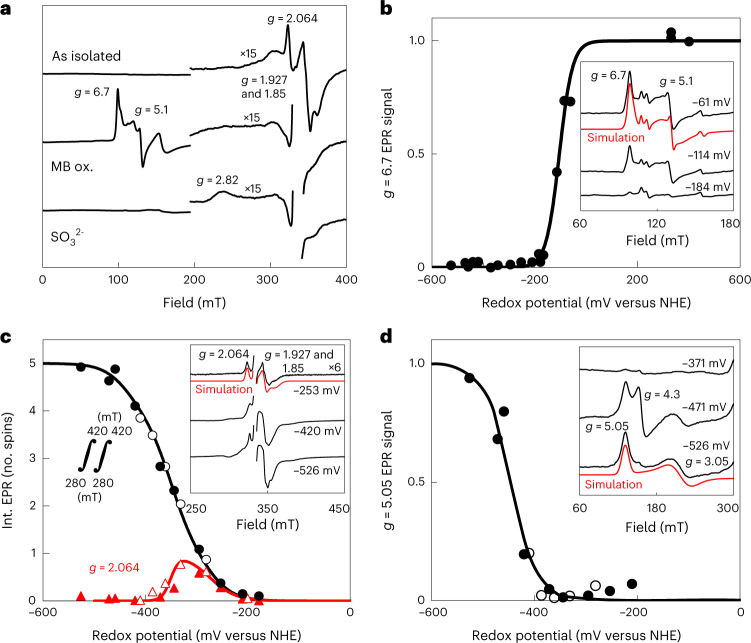
Determination of the redox potential of the metallocofactors in *Mt*Fsr via EPR spectroscopy. **a**, EPR spectra of as-isolated, methylene blue-oxidized (MB-ox.) and, consecutively, Na_2_SO_3_ (10 mM)-treated *Mt*Fsr. **b**–**d**, Dye-mediated redox titrations of indicated EPR signals (or double integral in **c**). Representative spectra at three selected potentials are shown in the insets, including *g* values and simulations (see text). EPR spectra for all samples are in Extended Data Fig. 6a,b,d. Nernst fits for *n* = 1 with *E*_m_ = −104 mV (**b**), −275, three times −350 and −435 mV (**c**) and −445 mV (**d**) are shown. NHE, normal hydrogen electrode. The fit for *g* = 2.064 used *n* = 1 (in red) for −275 mV and *n* = 2 for −350 mV (in black). EPR conditions: temperature, 10 K; modulation frequency, 100 kHz; modulation amplitude, 1.0 mT; microwave frequency 9.353 GHz; microwave power 20 mW except in **c**, where 0.2 mW. While one cluster indeed has a measured redox potential of −275 mV and three others are at −350 mV, one of them exhibits a lower potential of −435 mV. The presence of such a low redox potential cluster has already been seen in complex I and does not contradict our hypothesis regarding the electron flow.

**Extended Data Fig. 6:**
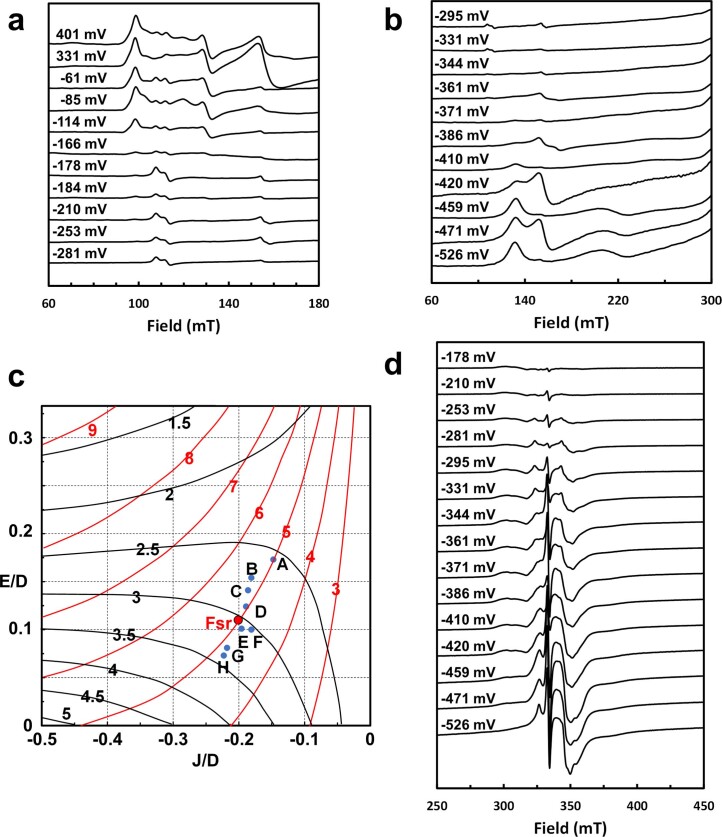
EPR spectra of the dye-mediated redox titrations of *Mt*Fsr and *g*-values as function of J/D and E/D as described by J. A. Christner et al. 1984. **a**, **b** and **d** The redox potentials at which samples were frozen are indicated. EPR intensities were scaled to correct for differences in concentration. EPR conditions: temperature, 10 K; modulation frequency, 100 kHz; modulation amplitude, 1.0 mT; microwave frequency, 9.353 GHz; microwave power, 20 mW (panel **d** 0.2 mW). **c**, Contours of the two highest *g*-values of the coupled ferrous siroheme-[4Fe-4S]^1+^ system as function of J/D and E/D according to Fig. 4 from^32^. The blue points are from *E. coli* sulfite reductase: A and B, KCl (two species); C, KF or KBr; E, urea; F, sodium formate; G, (Gdm)_2_SO_4_; H, KBr; D, spinach nitrite reductase; *Mt*FsR is shows as red point.

EPR spectroscopy at 10 K (Extended Data Fig. 6a–d) revealed that in as-isolated *Mt*Fsr high-spin (*S* = 5/2) and low-spin (*S* = 1/2) signals typical for the siroheme in sulfite reductases^27,28^ were absent, neglecting the sharp axial *S* = 5/2 EPR signal around *g* = 6, which, quantified by double integration of its simulation spectrum (*g* = 6.22, 5.92 and 1.98), is at most 3% of *Mt*Fsr. Apparently, on purification under strictly anaerobic conditions, the siroheme remains in its ferrous state. After methylene blue oxidation or on dye-mediated redox titration with *E*_m,7.5_ = −104 mV (all potentials refer to potentials versus the H_2_/H^+^ normal hydrogen electrode) an intense rhombic *S* = 5/2 EPR signal with *g* = 6.7 and 5.1 appeared (Fig. 4a,b). The spectrum could be simulated with three components: a main species with *g* = 6.70 and 5.10 (78%), a less abundant species (19%) with *g* = 6.80 and 5.08, but narrower linewidth, and the sharp axial *g* = 6 species already seen in as-isolated *Mt*Fsr. For both rhombic components *g* = 1.95 was taken as the third *g* value, as the experimental spectrum contained a weak [3Fe-4S]^1+^ signal from limited [4Fe‒4S]^2+^ breakdown upon oxidation. In sulfite reductase and other hemoproteins multiple high-spin species are common^29^. Addition of SO_3_^2−^ to methylene blue-oxidized *Mt*Fsr led to disappearance of the siroheme ferric high-spin signals and formation of a weak low-spin EPR signal, of which only the highest *g* value (2.8) was detectable, as in other sulfite reductases^30^.

In an enzyme approaching the complexity of the complex I, it is not feasible to determine all individual redox potentials of its five regular [4Fe‒4S]^1+/2+^ cubanes and the siroheme-bridged cubane. First, on the basis of distances in Fsr, extensive magnetic coupling^31^ between neighboring cubanes is anticipated, blurring individual EPR features. Second, the coupling between the ferrous siroheme and its cysteine-bridged reduced cubane leads to complex mixtures of sharp *g* = 1.94, broader *g* = 2.29 and very anisotropic *S* = 3/2 mimicking signals^32^. Third, we had to avoid sodium dithionite inherently containing SO_3_^2−^ and therefore used sodium borohydride-reduced F_420_, while following the solution potential with mediators. One [4Fe‒4S]^1+/2+^ cubane with simulated *g* values of 2.064, 1.927 and 1.85 was reduced at a relatively high potential and is also detected in as-isolated Fsr (Fig. 4a,c). From the amplitude of the second derivative of the experimental EPR spectrum at *g* = 2.064, *E*_m,7.5_ = −275 mV was estimated from fitting to the Nernst equation with n = 1 (Fig. 4c). The signal ‘disappeared’ on further reduction with *E*_m,7.5_ = −350 mV in a manner indicating cooperativity (*n* = 2). As super-reduction to [4Fe‒4S]^0^ is unlikely (*E*_m_ = −790 mV (ref. 33)), we interpret this phenomenon as reduction of two neighboring clusters of the *g* = 2.064 cluster. This cluster thus is number 2, 3 or 4′ (the siroheme cubane typically has a very low potential^27^). In the absence of sufficiently differing EPR features below −350 mV we double integrated the EPR spectra. On the basis of iron content divided by 24 (siroheme does not release Fe ions in acid) we quantified 4.5 ± 0.5 spin/subunit at the lowest attainable potential (−526 mV), which most likely corresponds to the five regular clusters. A fit for the spin integral as a function of the redox potential included the experimental *E*_m,7.5_ = −275 mV and *E*_m,7.5_ = −350 mV for both neighboring clusters. Avoiding overfitting, we could satisfactorily reproduce the data for five redox transitions with three midpoint potentials: one at *E*_m,7.5_ = −275 mV (experimental), one at a low potential to represent the lowest potential region (*E*_m,7.5_ = −435 mV) and three times *E*_m,7.5_ = −350 mV for the other three clusters (which includes the two clusters leading to broadening of the *g* = 2.064 signal).

In the low-field region, a species with unusual *g* values was detected (simulated *g* values 5.05, 3.05 and 1.96) at very low potential (Fig. 4d). It was accompanied in some samples by an isotropic *g* = 4.3 signal. But, since the integrated intensity was maximally 5% of the *g* = 5.05 species and non-Nernstian behavior was seen, it was not considered physiologically relevant. It has previously been shown that such a *g* = 5.05 species is not from a *S* = 3/2 system but from transitions of the siroheme–Fe^2+^ exchange coupled to [4Fe‒4S]^1+^ (J/D ≈ −0.2 and E/D ≈ 0.11, in which J, D and E are the effective Heisenberg exchange coupling parameter and the spin Hamiltonian zero-field splitting parameters of the spin quintet, respectively; Extended Data Fig. 6c)^32^. In full agreement with findings on the *Escherichia coli* assimilatory reductase^27^ a very low potential (*E*_m,7.5_ = −445 mV) was estimated.

### A prototypical sulfite reductase

**Fig. 5:**
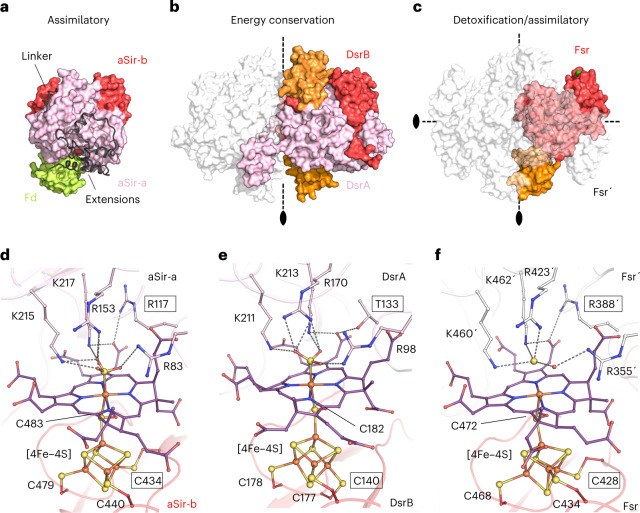
Overall structural comparison between aSir, dSir and Fsr. **a**–**c**, All structures are represented in surface, dimeric partners shown in white transparent and residues from the opposing monomer are labeled with a prime symbol. The black ovals and black dashed lines indicate the twofold symmetry axes. The inserted ferredoxin domains of DsrAB and *Mt*Fsr are colored in orange. **a**, aSir from *Zea mays* with its [2Fe‒2S] ferredoxin colored in light green (PDB 5H92). **b**, DsrAB from *A. fulgidus* (PDB 3MM5). **c**, *Mt*Fsr tetramer. For *Mt*Fsr, the green surface indicates the F_420_H_2_-oxidase position. **d**–**f**, Active site of sulfite reductases. Close-up of the active site and the functional siroheme surroundings in *E. coli* aSir (PDB 1AOP) (**d**), dSir of *A. fulgidus* (PDB 3MM5) (**e**) and *Mt*Fsr (**f**) in which HS^−^ was tentatively modeled. Residues coordinating the [4Fe‒4S] cluster, the siroheme and the sulfur species are shown as balls and sticks, while sulfur and iron are depicted as spheres. Framed residues highlight the differences between the siroheme‒[4Fe‒4S] binding in aSirs and dSirs.

**Extended Data Fig. 7:**
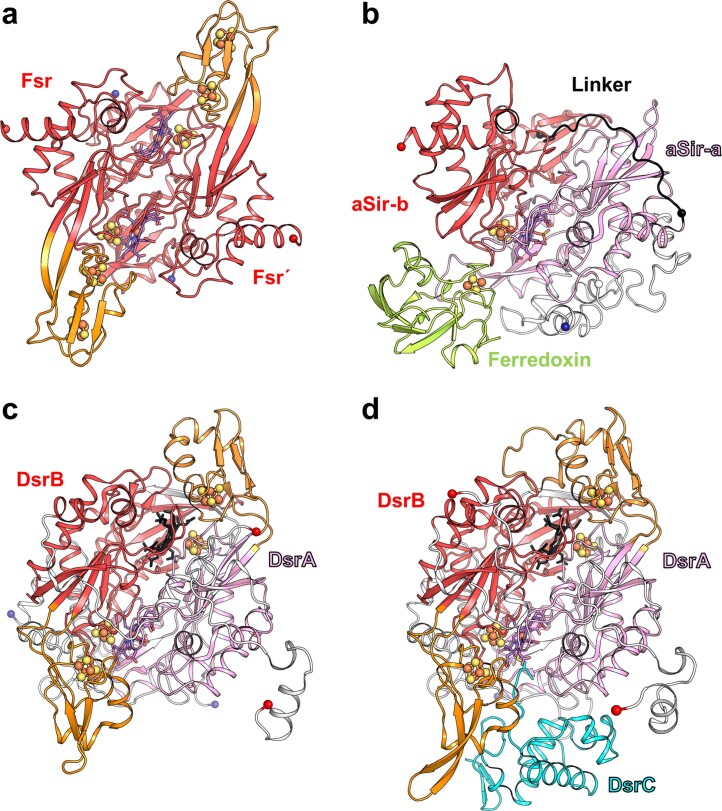
Overall structural comparison between Fsr, aSir and dSir. Cut-through view shown in cartoon of one dimer for Fsr and DsrAB. Ligands are shown as balls and sticks. **a**, The sulfite reductase domain with the inserted ferredoxin domain of *Mt*Fsr. Fsr´ corresponds to the opposite monomer. **b**, aSir from *Zea mays* and its [2Fe-2S]-ferredoxin coloured in light green (PDB 5H92). **c**, DsrAB from *A. fulgidus* (PDB 3MM5) and **d**, DsrABC from *D. vulgaris* (PDB 2V4J). The inserted ferredoxin domains of Fsr, DsrA and DsrB are coloured in orange. The catalytic siroheme in DsrAB is coloured in purple and the structural siroheme is coloured in black. DsrAB from *D. vulgaris* contains sirohydrochlorin instead of siroheme.

The C-terminal domain of Fsr represents the simplest sulfite reductase crystallized so far. While Fsr shares the common fold of sulfite reductases (Extended Data Fig. 7a and Supplementary Fig. 6)^9,13,14^, it lacks the large N- and C-terminal extensions found in aSirs and dSirs, which presumably serve to strengthen dimerization and to interact with partners^34^ (Fig. 5a–c). Without these extensions, Fsr is much more compact—possibly a thermophilic trait. Each Fsr protomer contains one functional siroheme center. In comparison, dSirs harbor one functional and one structural siroheme center in each DsrAB heterodimer, while aSirs have lost one siroheme‒[4Fe‒4S] site (Extended Data Fig. 7b–d).

Although Fsr is phylogenetically more distant from aSirs than from dSirs, it superposes well with the first and second halves of aSirs (Supplementary Figs. 6–10). The position of the C terminus of Fsr coincides with the beginning of the linker connecting the two half domains in aSirs (Extended Data Fig. 7a,b and Supplementary Figs. 7 and 8). This detail corroborates the theory that modern aSirs evolved by duplication and fusion events.

The inserted ferredoxin domain in Fsr is at the same position as the ferredoxin domain in DsrA or DsrB (Extended Data Fig. 7a,c,d and Supplementary Figs. 9 and 10). There is a remarkable three-dimensional conservation of the electron connectors between Fsr, DsrA, DsrB and even the aSir from *Zea mays*, where the external [2Fe‒2S] ferredoxin sits on the core of the sulfite reductase^35^ (Fig. 5a–c). Such a conserved position suggests a common origin, but could also be due to the restricted access of the [4Fe–4S]–siroheme and the selection pressure towards an optimized distance for electron transfer.

### Fsr has traits of assimilatory sulfite reductases

**Extended Data Fig. 8:**
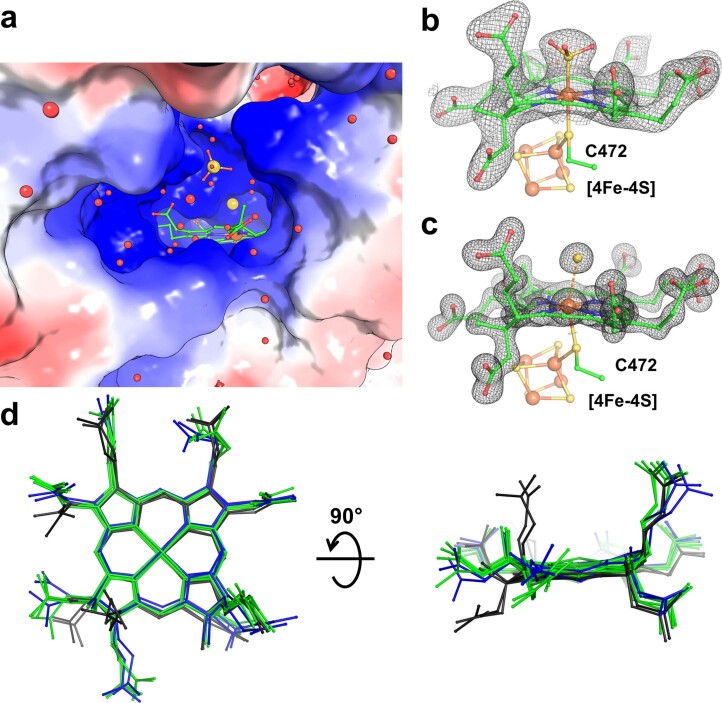
Siroheme conformation within Fsr. **a**, Electrostatic charge profile of *Mt*Fsr shown in surface is coloured in red and blue to represent acidic and basic patches, respectively. The siroheme is accessible via a positively charged solvent channel. Carbon, oxygen, nitrogen, sulfur and iron are coloured in green, red, blue, yellow and orange, respectively. **b** and **c**, Close up of the axial ligands bound on the siroheme of *Mj*Fsr (b) and *Mt*Fsr (c). The 2*F*_o_-*F*_c_ map of the siroheme and SO_3_^2−^ are contoured to 1.5-σ in *Mj*Fsr, while the siroheme and HS^−^ is contoured to 3-σ in *Mt*Fsr. In *Mj*Fsr the Fe-siroheme is equidistant (2.3 Å) to the sulfur from the modelled SO_3_^2−^ and the bridging-sulfur of the cysteine 472, suggesting a tight covalent binding. In *Mt*Fsr, the bridging-sulfur of the cysteine 472 is at a distance of 2.6 Å to the Fe-siroheme and the sulfur from the modelled HS^−^ is 2.9 Å distant to the Fe-siroheme, indicating a loose binding of the HS^−^, which might result from a reduction event by X-ray radiation^60^. **d**, Siroheme superposition between aSirs (1AOP, 5H92), dSirs (3MM5, 2V4J) and Fsrs. Siroheme from aSirs and Fsr are coloured in green, structural siroheme/sirohydrochlorin from dSirs in black and dSirs functional sirohemes in blue. Superposition analysis shows that the functional sirohemes are arranged in a highly similar manner, whereas the conformation of the structural siroheme or sirohydrochlorin differ, which highlights the strong influence of the protein environment on the siroheme geometry.

While the sirohemes of DsrAB are partially surface exposed to interact with DsrC (Extended Data Fig. 1)^13^, the Fsr sirohemes are buried but still accessible via a positively charged solvent channel (Extended Data Fig. 8). As in DsrAB, the two sirohemes within one Fsr dimer are in close proximity (9.4 Å; Supplementary Fig. 11)^14^.

The binding of the siroheme in *Mj*Fsr and *Mt*Fsr is highly conserved. It is mainly anchored by positively charged residues from one protomer, while the dimeric partner binds the adjacent [4Fe‒4S] cluster establishing the siroheme‒[4Fe‒4S] center, as reported for other sulfite reductases^14^. On the basis of the observed electron density, we tentatively modeled a SO_3_^2−^ bound to the siroheme iron (2.3 Å; Extended Data Fig. 8b) in *Mj*Fsr. In *Mt*Fsr, the axial ligand is a single atomic species at all sites of the asymmetric unit, which is in proximity but not covalently bound to the iron (2.9 Å; Extended Data Fig. 8c). The anion HS^−^ was modeled in the electron density based on the pH 5.5 in the crystallization solution. This species could be the result of cocrystallizing Fsr with reduced F_420_, which might have forced the complete reduction of bound SO_3_^2−^.

In *Mj*Fsr, four positively charged residues (Arg 355, Arg 423, Lys 460 and Lys 462), which are perfectly conserved across sulfite reductases (Fig. 5d,e and Supplementary Figs. 7 and 9), bind the SO_3_^2−^ and two water molecules. In *Mt*Fsr, the modeled HS^−^ is bound by Arg 423, Lys 460 and Lys 462, and one water molecule is stabilized by Arg 355 (Fig. 5f). Group II Fsr found in the genome of anaerobic methanotrophic archaea^6^ (except for ‘*Candidatus* *Methanoperedens nitroreducens*’) and *Methanosarcinales*, should have a larger binding pocket and two arginines of Group I Fsr are replaced by a lysine and glycine. This suggests that the functionally uncharacterized Group II Fsr has a different substrate specificity^6,17^. Interestingly, the second isoform found in *M. thermolithotrophicus* harbors one arginine but exchanged the other one for a threonine (Thr 438; Supplementary Fig. 2), indicating an alternative physiological function.

The active site of Fsr shows the same traits as an assimilatory sulfite reductase: an arginine at position 388, and the coordination of the siroheme-coupled [4Fe‒4S] cluster by the canonical motif (CX_5_CX_*n*_CX_3_C; Fig. 5d,f). In comparison, DsrAs contain a conserved threonine where aSirs have arginine (αThr 136 in *Desulfovibrio vulgaris* and αThr 133 in *Archaeoglobus fulgidus*) and the catalytically active [4Fe‒4S] cluster coupled to the siroheme of DsrBs is coordinated by the canonical motif CX_*n*_CCX_3_C (Fig. 5e). Fsr must therefore follow the same catalytic path as aSirs; the six-electron reduction of SO_3_^2−^ to S^2−^ should be unidirectional, without the formation or consumption of intermediates (for example, thiosulfate or trithionate). *Mt*Fsr did not accept thiosulfate as an electron acceptor, which is in agreement with the findings for *Mj*Fsr^5^. We also monitored F_420_-reduction by *Mt*Fsr with S^2−^ as substrate (up to 10 mM) and observed no reaction. The addition of 10 mM S^2−^ to 1.4 mM of Na_2_SO_3_ also had no effect on the F_420_H_2_ oxidation rate. Taken together, these results support that Fsr indeed acts like an aSir.

On the basis of its equal *V*_max_ but six-fold lower *K*_m_ value (Table 1), *Mt*Fsr prefers NO_2_^−^ over SO_3_^2−^, a property that may expand its role from sulfite detoxification to ammonium production, as *M. thermolithotrophicus* has been reported to grow on nitrate as a sole source of nitrogen^36^. If the archaeon uses a nitrate reductase, NO_2_^−^ would accumulate and Fsr would be a suitable candidate for NO_2_^−^ conversion. In addition, we have shown that *Mt*Fsr reduces selenite (SeO_3_^2−^) in vitro with a relative activity of 20.7 ± 7.5% compared to SO_3_^2−^ (see Methods). These promiscuous activities could expand the physiological range of the enzyme, but also its biotechnological applications.

**Table 1:** Kinetic parameters of *Mt*Fsr and *Mj*Fsr (mean ± s.d., *n* = 3 independent experiments)

Enzyme	Substrate	Apparent *K*_m_ (µM)	Apparent *V*_max_ (µmol of F_420_H_2_ oxidized min^−1^ mg^−1^ of Fsr)
*Mj*Fsr, from ref. 5	SO_3_^2−^	12.2 ± 1	16
*Mt*Fsr	SO_3_^2−^	15.6 ± 2.0	27.6 ± 0.9
*Mt*Fsr	NO_2_^−^	2.5 ± 0.2	27.2 ± 0.5

Source data

## Discussion

Some methanogens show a remarkable tolerance to SO_3_^2−^, one of the sulfur-reactive species that can cause oxidative damage to the methanogenic machinery. Besides the possibility that those methanogens can keep low intracellular SO_3_^2−^ concentrations through pumping mechanisms, the cytoplasmic Group I Fsr is used as a first line of defense to convert toxic SO_3_^2−^ into HS^−^, which can then be used for sulfur assimilation. The efficient SO_3_^2−^ detoxification strategy of *Methanococcales* relies on the enormous amount of expressed Fsr, which constitutes 5–10% of the cellular protein (Extended Data Fig. 2b,c and Methods), but also on the use of abundant F_420_H_2_, which can be rapidly regenerated via H_2_ oxidation by Frh^22^.

Fsr discloses a ‘cofactor swapping’ between two subunits forming a homodimer in a head-to-tail configuration, which dimerizes with a second homodimer, creating a butterfly-shaped tetramer. As a result, the centrally located sulfite reductase domains are surrounded by F_420_H_2_-oxidase domains. These shuttle electrons via three [4Fe‒4S] cluster from one subunit to the other two [4Fe‒4S] cluster and the siroheme‒cysteine‒[4Fe‒4S] cofactor of the other subunit within the functional dimer. In contrast to the bidirectional hydrogenase Frh, which maintains an isopotential of *E*′^0^ ≈ −400 mV (ref. 25), the different metalloclusters of Fsr must establish a downhill redox potential from the FAD to the siroheme‒[4Fe‒4S]. Our electrochemical and spectroscopic studies indicate that the electrons carried by F_420_H_2_ are immediately transferred to the siroheme‒[4Fe‒4S] (Fig. 4a,b and Extended Data Fig. 6a). The metallocofactors should ensure efficient electron transfer rather than serving as a transient storage, and a cascade of redox potential from −380 mV (F_420_/F_420_H_2_ redox potential under certain physiological conditions^22^) to −116 mV (*E*´^0^ of HSO_3_^−^/HS^−^) is expected.

Once reduced, the siroheme‒[4Fe‒4S] could transfer the electrons to the sulfur species covalently bound to its Fe. dSirs physiologically perform a two-electron reduction to allow the transfer of the sulfur intermediate to DsrC. In contrast, aSirs and Fsr perform a three times two-electron reduction to release HS^−^. A positively charged environment around the active site attracts SO_3_^2−^ and an organized water network has been proposed to provide fast proton transfer via the Grotthuss mechanism, allowing successive SO_3_^2−^ reduction (Extended Data Fig. 8a)^16,37^. Despite a strikingly similar position of the residues involved in substrate binding, aSirs/Fsr and dSirs react differently. With the possibility of genetically modifying *M. maripaludis* or *M. jannaschii*, it would be worthwhile to exchange the residues that confer aSir traits at the active site (Arg 388, Cys 428) with dSir ones and observe the effects on the phenotype^7,8^.

**Extended Data Fig. 9:**
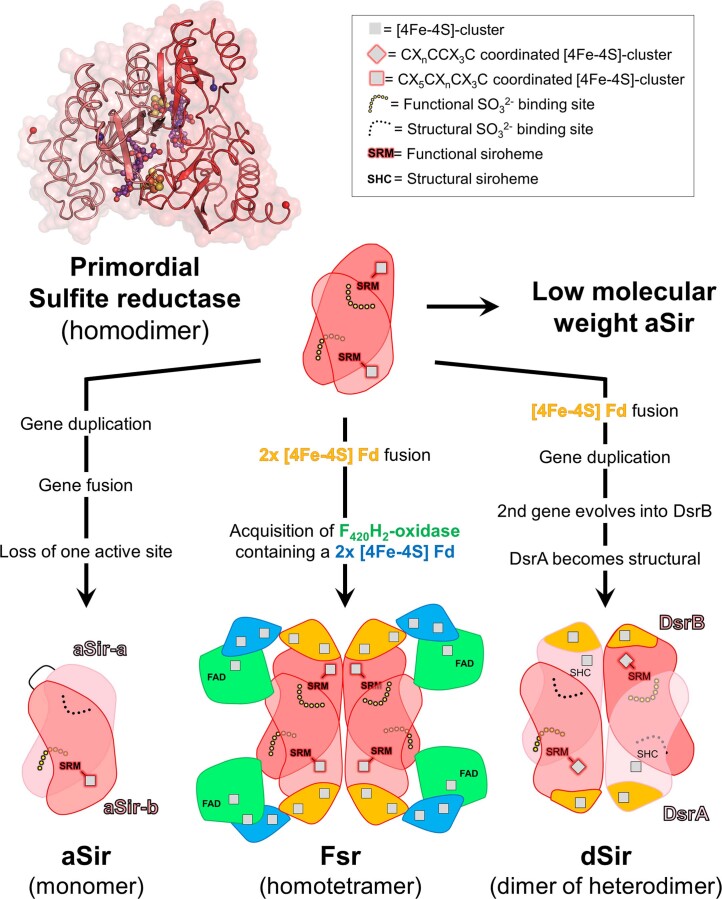
Theoretical evolutionary scenario of sulfite reductases. The proposed route is based on the assumption that aSir, dSir and Fsr could have evolved from a common ancestor. The primordial sulfite reductase model corresponds to the elementary sulfite reductase core of the *Mt*Fsr structure. The different steps that led to the evolution of this progenitor to modern Fsr can be hypothesized based on its modular organization. In a straightforward and simple model, a ferredoxin with 2 × [4Fe‒4 S]-cluster could have been inserted into the elementary sulfite reductase module. Then an F_420_H_2_-oxidase with a ferredoxin domain (Fqo/FpoF-like) would have been fused to the N-terminus of the sulfite reductase domain containing the inserted ferredoxin. Some members of the Sir superfamily might have arisen from one of these steps^18^. Such a hypothesis is exemplified by the similarities between the quaternary organization of Fsr and DsrAB and the active site of Fsr and aSir.

Throughout evolution, sulfite reductases have been kept to detoxify SO_3_^2−^ as well as to conserve energy by dissimilatory SO_3_^2−^reduction or oxidation of H_2_S^38^. Based on sequence and structural similarity with enzymes from different superfamilies, it has been proposed that modern sulfite reductases originated from a primordial Sir/Nir that functioned as a self-complementary homodimer^18^. A snapshot of this progenitor can be derived from the Fsr structure, as the organization of its sulfite reductase domain is highly simplified (Extended Data Fig. 9). The evolution of Fsr is still a matter of debate but it needs to be thoroughly studied, as its discovery has reinforced the question of whether sulfate respiration or methanogenesis was the primeval means of energy conservation during the evolution of early Archaea^39,40^. Both metabolisms, related to each other, possibly coexisted or even coexist still^6,18,41^. Methanogens might have lost the genes required for complete sulfate dissimilation over time, but kept the sulfite reductase to adapt to environments where SO_3_^2−^ fluctuations do occur. However, *M. thermolithotrophicus* appears to use a complete sulfate-reduction pathway, as it is able to grow on sulfate as its sole sulfur source^4^. This assimilation pathway requires SO_3_^2−^ as an intermediate, and Fsr is expected to orchestrate its reduction. Although further studies need to investigate whether this methanogen can also express other enzymes of the sulfate-reduction pathway, the structural elucidation of Fsr provides the first snapshot of a sulfate reduction-associated enzyme in a methanogen.

## Methods

### Methanogenic archaea strains and cultivation medium

*M. jannaschii* (DSM 2661) and *M. thermolithotrophicus* (DSM 2095) cells were obtained from the Leibniz Institute DSMZ-German Collection of Microorganisms and Cell Cultures (Braunschweig) and cultivated in a previously described minimal medium with some modifications^42^.

### Reagents used for this study

Lists of reagents and providers are provided in Supplementary Table 1.

### Sulfur-free cultivation medium for *Methanococcales*

Per liter of medium: 558 mg KH_2_PO_4_ (final concentration 4.1 mM), 1 g KCl (13.4 mM), 25.13 g NaCl (430 mM), 840 mg NaHCO_3_ (10 mM), 368 mg CaCl_2_·2H_2_O (2.5 mM), 7.725 g MgCl_2_·6H_2_O (38 mM), 1.18 g NH_4_Cl (22.06 mM), 61.16 mg nitrilotriacetic acid (0.32 mM), 6.16 mg FeCl_2_·4H_2_O (0.031 mM), 10 µl 2 mM Na_2_SeO_3_·5H_2_O stock (0.02 µM), 3.3 mg Na_2_WO_4_·2H_2_O (0.01 mM) and 2.42 mg Na_2_MoO_4_·2H_2_O (0.01 mM) were dissolved under constant stirring in a measuring cylinder with 750 ml of deionized H_2_O (dH_2_O)^42^. Resazurin (1 ml, 1.5 mM) was added (0.0015 mM) and 10 ml of sulfur-free trace elements (see below) were added subsequently. For *M. jannaschii*, 30.24 g PIPES (100 mM final) was used as a buffer and a pH 7.0 was adjusted using sodium hydroxide pellets. For *M. thermolithotrophicus* the pH was set to either 7.6 with 50 mM Tris–HCl as buffer or to 6.2 with 50 mM MES. The media were filled up to a final volume of 1 liter by the addition of dH_2_O.

The cultivation media were transferred in a 1 l pressure-protected Duran laboratory bottle with a magnetic stirring bar. The Duran flask was closed with a butyl rubber stopper and degassed by applying 3 min of evacuation, followed by 30 seconds of ventilation with 1 × 10^5^ Pa N_2_ atmosphere, under constant magnetic stirring. This was repeated 15 times and at the final ventilation step an overpressure of 0.3 × 10^5^ Pa N_2_ was applied.

### Trace element composition

A 100-fold-concentrated trace element solution was prepared by first dissolving 1.36 g nitrilotriacetic acid (7.1 mM) in 800 ml dH_2_O under magnetic stirring. The pH was shifted to 6.2 by adding NaOH pellets. Then, 89.06 mg MnCl_2_·4H_2_O (0.45 mM), 183.3 mg FeCl_3_·6H_2_O (0.68 mM), 60.27 mg CaCl_2_·2H_2_O (0.41 mM), 180.8 mg CoCl_2_·6H_2_O (0.76 mM), 90 mg ZnCl_2_ (0.66 mM), 37.64 mg CuCl_2_ (0.28 mM), 46 mg Na_2_MoO_4_·2H_2_O (0.19 mM), 90 mg NiCl_2_·6H_2_O (0.38 mM) and 30 mg VCl_3_ (0.19 mM) were added separately. The trace element mixture was filled up to a final volume of 1 liter with dH_2_O.

### Anaerobic growth of *Methanococcales*

For all studied archaea, cell growth was measured spectrophotometrically by measuring the optical density at 600 nm (OD_600_). To control the purity of the culture, samples were taken and analyzed via light microscopy. Both methanogens were cultivated at 65 °C, unless stated otherwise, with 1 × 10^5^ Pa of H_2_/CO_2_ in the gas phase. *M. jannaschii* was cultivated in flasks and *M. thermolithotrophicus* was cultivated in flasks or a fermenter.

### Growth of *M. jannaschii*

Duran bottles (10× 1 liter) were sealed with butyl rubber stoppers and the gas phase was exchanged for H_2_/CO_2_ (80:20, 1 × 10^5^ Pa). A 100-ml portion of anaerobic cultivation media was transferred into each bottle (ratio 1:10 of medium/gas phase), with 1 mM Na_2_SO_3_ as a sole sulfur source. A portion of 5 ml of overnight culture (OD_600_ of 0.9) was used as an inoculum for 100 ml media. No additional reductant was added. The cultures were placed at 65 °C, with standing for at least one hour, followed by overnight shaking at 180 rotations per minute without light. The cells were collected in exponential phase with a final OD_600_ of 1.83 by immediately transferring them in an anaerobic tent (N_2_/CO_2_ atmosphere at a ratio of 90:10), followed by anaerobic centrifugation for 30 min at 6,000*g* at 4 °C. The cell pellet was transferred in a sealed bottle gassed with 0.3 × 10^5^ Pa N_2_ and flash frozen in liquid N_2_ to be stored at −80 °C.

### Growth of *M. thermolithotrophicus* for Fsr crystallization

*M. thermolithotrophicus* was grown in a fermenter at 50 °C with 10 mM sulfate (SO_4_^2−^) as sole sulfur substrate. Since SO_3_^2−^ could be an intermediate in the SO_4_^2−^ reduction pathway it would require the expression of Fsr. Therefore, 1.5 l of anaerobic cultivation medium with 10 mM SO_4_^2−^ were continuously bubbled with H_2_ and CO_2_ (80:20, 2 × 10^4^ Pa) and inoculated with 100 ml preculture (OD_600_ of 4.2). Since the fermenter is an open system, we set a more alkaline pH (7.6) to prevent evaporation of produced S^2−^. Here, it should predominantly be present in the form of HS^−^, and not H_2_S, and therefore stay for longer time in the medium. The pH was checked every two hours by using a pH indicator. The cells were grown until late exponential phase (OD_600_ of 2.97) and then immediately transferred in an anaerobic tent (N_2_/CO_2_ atmosphere at a ratio of 90:10). Cells were collected by anaerobic centrifugation for 30 min at 6,000*g* at 4 °C. A 1.5-l culture with an OD_600_ of 2.97 yielded 19.25 g of cells (wet weight). The cell pellet was transferred in a sealed bottle, gassed with 0.3 × 10^5^ Pa N_2_, flash frozen in liquid N_2_ and stored at −80 °C.

### Growth of *M. thermolithotrophicus* for Fsr activity assays

To perform enzymatic activity assays, *M. thermolithotrophicus* was directly grown on 2 mM Na_2_SO_3_. The ten 1-l Duran bottles were sealed with butyl rubber stoppers and the gas phase was exchanged for H_2_ and CO_2_ (80:20, 1 × 10^5^ Pa). A 100 ml of anaerobic cultivation media containing 50 mM MES at pH 6.2 was transferred in each bottle (ratio of 1:10 of medium/gas phase), with 2 mM Na_2_SO_3_ final as a sole sulfur source. A 5-ml portion of overnight-grown culture (OD_600_ of 1.7) was used as an inoculum for 100 ml of media. No additional reductant was added. The cultures were placed at 65 °C, with standing overnight. The cells were grown until early exponential phase (OD_600_ of 0.8), since we assumed that most SO_3_^2−^ has not been converted into HS^−^ yet and that Fsr should be highly expressed and active. The cells were immediately collected by transferring them in an anaerobic tent (N_2_/CO_2_ atmosphere at a ratio of 90:10), followed by anaerobic centrifugation for 30 min at 6,000*g* at 4 °C. The cell pellet was transferred in a sealed bottle, gassed with 0.3 × 10^5^ Pa N_2_, flash frozen in liquid N_2_ and stored at −80 °C.

### Sulfite growth inhibition

*M. thermolithotrophicus* was grown on different Na_2_SO_3_ concentrations to determine the growth-inhibiting threshold. For this, 250-ml serum flasks were sealed with a butyl rubber stopper and the gas phase was exchanged for H_2_ and CO_2_ (80:20, 1 × 10^5^ Pa). A 10-ml portion of anaerobic cultivation media with a pH set at 6.2 with 50 mM MES was transferred into each bottle. Then, different Na_2_SO_3_ concentrations (2 mM, 10 mM, 20 mM, 30 mM and 40 mM final) were added in triplicate as a sole sulfur source, and 2 mM Na_2_S was used as a control. The cultures grew at 65 °C for 22 hours, with standing. The three biological replicates for each setup are represented as dots in Extended Data Fig. 2a, with the standard deviation shown as bars.

### Growth of *M. thermolithotrophicus* for titrations and EPR spectroscopy

Due to the high demand of *Mt*Fsr for titration and EPR spectroscopy experiments, *M. thermolithotrophicus* was grown in one 10-l fermenter with SO_4_^2−^ as a sole sulfur substrate and in another 10-l fermenter with SO_3_^2−^ as a sole sulfur source, to boost *Mt*Fsr natural expression. The fermenter containing SO_4_^2−^ was performed as described above with an inoculum of 350 ml (OD_600_ of 3.2). A 7.4-l culture with an OD_600_ of 4.8 yielded 74 g of cells (wet weight). In the SO_3_^2−^ fermenter, *M. thermolithotrophicus* was grown at 50 °C in 7 l anaerobic cultivation medium with a pH of 6.2 supplemented with 5 mM SO_3_^2−^ as a sole sulfur substrate, continuously bubbled with H_2_ and CO_2_ (80:20, 2 × 10^4^ Pa). A 600-ml preculture (OD_600_ of 2.34) was used as inoculum. The cells were grown until an OD_600_ of 2.48 and then immediately transferred in an anaerobic tent (N_2_/CO_2_ atmosphere at a ratio of 90:10). Cells were collected by anaerobic centrifugation for 30 min at 6,000*g* at 4 °C and a final yield of 51 g of cells (wet weight) was obtained. The cell pellets were transferred in a sealed bottle, gassed with 0.3 × 10^5^ Pa N_2_, flash frozen in liquid N_2_ and stored at −80 °C.

### Genome sequencing of *M. thermolithotrophicus*

*M. thermolithotrophicus* was anaerobically grown in the above-described medium and 2 mM Na_2_S was used as a sulfur source. A total culture volume of 20 ml was used. Cells were aerobically collected by centrifugation (30 min, 6,000*g* at 4 °C). DNA was extracted and purified based on ref. 43. Quality control, library preparation and sequencing (PacBio Sequel II) were performed in the Max Planck-Genome-Centre (Cologne).

### Purification of Fsr

All steps were performed under the strict exclusion of oxygen and daylight. Protein purifications were carried out in a Coy tent with an N_2_ and H_2_ atmosphere (97:3) at 20 °C under yellow light. For both Fsr, three to five chromatography steps were used with some variations. Fsr purification was further followed via activity assays and on the basis of absorbance peaks at wavelengths of 280, 420 and 595 nm. Each elution profile was systematically controlled by SDS–PAGE to select the purest fractions.

### Purification of *Mj*Fsr

*M. jannaschii* cells (13.5 g wet weight) were thawed under warm water and transferred in an anaerobic tent (N_2_/CO_2_ atmosphere at a ratio of 90:10). Cells were diluted by three volumes of lysis buffer (50 mM Tricine/NaOH pH 8.0, 2 mM dithiothreitol (DTT)) and disrupted by sonication: 7 cycles at 62% intensity with 30 pulses followed by 1 min break (probe MS76, SONOPULS Bandelin). Cell debris was removed anaerobically via centrifugation (21,000*g*, one hour, room temperature). The protein concentration (measured by Bradford) of the supernatant was estimated to 4.68 mg ml^−1^. The supernatant was transferred to a Coy tent (N_2_/H_2_ atmosphere of 97:3) under yellow light at 20 °C. The sample was diluted with two volumes of lysis buffer and passed through a 0.2-µm filter (Sartorius). The filtered sample was loaded on a 10-ml Q Sepharose high-performance column (GE Healthcare), which was previously equilibrated with 5 column volumes (CV) of lysis buffer. The column was then washed with 2 CV of lysis buffer. *Mj*Fsr was eluted by a gradient of NaCl (from 0.1 to 0.6 M) in 27 CV at a flow rate of 1.5 ml min^−1^ in fraction sizes of 3.5 ml. *Mj*Fsr eluted between 0.37 and 0.41 M NaCl. The fractions of interest were pooled and 1:1 diluted with HIC buffer (25 mM Tris–HCl pH 7.6, 2 M (NH_4_)_2_SO_4_ and 2 mM DTT). The sample was filtered and applied to a Source15Phe 4.6/100 PE column (GE Healthcare) previously equilibrated with the HIC buffer. The column was then washed with 2 CV of 25 mM Tris–HCl pH 7.6, 1.4 M (NH_4_)_2_SO_4_ and 2 mM DTT buffer. The elution was performed at a flow rate of 0.8 ml min^−1^ by a decreasing gradient of (NH_4_)_2_SO_4_ (1.4 to 0 M) over 90 min, with a fractionation size of 2 ml. Fsr eluted in the fractions at 0.9 to 0.78 M (NH_4_)_2_SO_4_. Those fractions were merged and concentrated using a 30-kDa-cutoff filter (Merck Millipore). The concentrated sample was passed through a 0.2-µm filter and injected on a Superdex 200 Increase 10/300 GL (GE Healthcare) equilibrated in storage buffer (25 mM Tris–HCl pH 7.6, containing 10% v/v glycerol and 2 mM DTT). The elution was performed at a flow rate of 0.4 ml min^−1^ in the storage buffer. *Mj*Fsr eluted as a sharp Gaussian peak at 10.4 ml. The pooled samples were concentrated by passing them through a 30-kDa-cutoff filter, and the final concentration was measured by the Bradford method (BioRad). The sample was immediately crystallized at a concentration of 6.1 mg ml^−1^.

### Purification of *Mt*Fsr for crystallization

Cells (19.25 g wet weight) derived from a fermenter were thawed under warm water and transferred to an anaerobic tent containing an atmosphere of N_2_/CO_2_ (90:10). Cells were lysed by osmotic shock through the addition of 60 ml lysis buffer (50 mM Tricine/NaOH pH 8.0, 2 mM DTT). Cell lysate was homogenized by sonication: 3 cycles at 70% intensity with 30 pulses followed by 1 min break (probe MS76, SONOPULS Bandelin) and cell debris was removed anaerobically via centrifugation (21,000*g*, one hour at 4 °C). The supernatant was transferred in a Coy tent (N_2_/H_2_ atmosphere of 97:3), with yellow light at 20 °C. The sample was filtered through a 0.2-µm filter (Sartorius) and was passed onto a DEAE fast-flow column (30 ml), equilibrated with lysis buffer. The column was then washed with 2 CV of lysis buffer. *Mt*Fsr was eluted with a gradient of 0.1 to 0.6 M NaCl in 120 min at a flow rate of 2.5 ml min^−1^ and in fractionation sizes of 4 ml. *Mt*Fsr eluted between 0.3 and 0.39 M NaCl. The fractions of interest were merged, diluted by 3 volumes of lysis buffer and filtered through a 0.2-µm filter. The filtered sample was loaded on a 15-ml Q Sepharose high-performance column, equilibrated with lysis buffer. The column was washed with 2 CV of lysis buffer. A gradient of 0.15 to 0.55 M NaCl in 120 min with a flow rate of 1 ml min^−1^ was performed and fractions of 1.5 ml were collected. *Mt*Fsr eluted between 0.49 and 0.53 M NaCl. Fractions of interest were pooled and diluted with 2 volumes of HAP buffer (20 mM K_2_HPO_4_/HCl pH 7.0 and 2 mM DTT) and subsequently filtered through a 0.2-µm filter. The filtered sample was applied to a 10-ml hydroxyapatite column type 1 (Bio-Scale Mini CHT cartridges, BioRad) equilibrated with HAP buffer. The column was washed with 2 CV of HAP buffer and a gradient of 0.02 to 0.5 M K_2_HPO_4_ for 60 min at a flow rate of 2 ml min^−1^ was performed and 3-ml fractions were collected. *Mt*Fsr eluted between 0.28 and 0.39 M K_2_HPO_4_ and the respective fractions were pooled. The pool was diluted 1:3 with 25 mM Tris–HCl pH 7.6, 2 M (NH_4_)_2_SO_4_ and 2 mM DTT (HIC buffer). The filtered sample was applied to a Source15Phe 4.6/100 PE column (GE Healthcare) previously equilibrated with the HIC buffer. The column was then washed with 2 CV of HIC buffer. A gradient of (NH_4_)_2_SO_4_ ranging from 2 to 1 M was performed for 30 min at a flow rate of 0.8 ml min^−1^ with a fractionation size of 1 ml. *Mt*Fsr eluted between 1.38 and 1.23 M (NH_4_)_2_SO_4_ and the respective fractions were pooled. The buffer was exchanged for the storage buffer (25 mM Tris–HCl pH 7.6, containing 10% v/v glycerol and 2 mM DTT) by using a 30-kDa-cutoff filter (6 ml, Merck Millipore) and *Mt*Fsr was concentrated to 11.06 mg ml^−1^ in a volume of 120 µl. The protein concentration was estimated by the Bradford method. The sample was immediately crystallized.

### Purification of *Mt*Fsr for enzyme activity assays

SO_3_^2−^-grown cells (8 g wet weight) were thawed under warm water and transferred to an anaerobic tent containing an atmosphere of N_2_/CO_2_ (90:10). Cells were lysed by osmotic shock through the addition of 60 ml lysis buffer (50 mM Tricine/NaOH pH 8.0, 2 mM DTT). Cell lysate was homogenized by sonication: 9 cycles at 75% intensity with 30 pulses followed by 1 min break (probe KE76, SONOPULS Bandelin) and cell debris was removed anaerobically via centrifugation (21,000*g*, one hour at 4 °C). The supernatant was transferred to a Coy tent (N_2_/H_2_ atmosphere of 97:3) under yellow light at 20 °C and was diluted with 90 ml lysis buffer, filtered through a 0.2-µm filter. The filtered sample was applied to a 10-ml DEAE fast-flow column (GE Healthcare), which was previously equilibrated with lysis buffer. The column was then washed with 2 CV of lysis buffer. A gradient of 0.1 to 0.6 M NaCl was applied for 120 min at a flow rate of 2.5 ml min^−1^ and fractions of 4 ml were collected. *Mt*Fsr eluted between 0.34 and 0.4 M NaCl. The fractions of interest were merged and diluted by 3 volumes of lysis buffer. The filtered sample was loaded on a 10-ml Q Sepharose high-performance column (GE Healthcare) and a gradient of 0.15 to 0.55 M NaCl was applied for 120 min with a flow rate of 1 ml min^−1^. Fractions of 1.5 ml were collected. *Mt*Fsr eluted between 0.49 and 0.53 M NaCl. The *Mt*Fsr fractions were pooled, and three times diluted with HAP buffer (20 mM K_2_HPO_4_/HCl pH 7.0 and 2 mM DTT). The filtered sample was applied to a 10-ml hydroxyapatite type 1 (Bio-Scale Mini CHT cartridges, BioRad) equilibrated with HAP buffer. The column was washed with 2 CV of HAP buffer and a gradient of 0.02 to 0.5 M K_2_HPO_4_ in 60 min at a flow rate of 2 ml min^−1^ was performed. Fraction sizes of 1.5-ml were collected. *Mt*Fsr eluted between 0.25 and 0.42 M K_2_HPO_4_ and the respective fractions were pooled. The pool was diluted with 3 volumes of HIC buffer (25 mM Tris–HCl pH 7.6, 2 M (NH_4_)_2_SO_4_ and 2 mM DTT). The filtered sample was applied to a Source15Phe 4.6/100 PE column (GE Healthcare) previously equilibrated with the HIC buffer. The column was then washed with 2 CV of 25 mM Tris–HCl pH 7.6, 1.6 M (NH_4_)_2_SO_4_ and 2 mM DTT buffer. *Mt*Fsr was eluted in a gradient of 1.6 to 0.8 M of (NH_4_)_2_SO_4_ in 25 min at a flow rate of 0.8 ml min^−1^ and a fractionation size of 1 ml. *Mt*Fsr eluted between 1.43 and 1.28 M (NH_4_)_2_SO_4_ and the respective fractions were pooled. The buffer was exchanged for the storage buffer (25 mM Tris–HCl pH 7.6, containing 10% v/v glycerol and 2 mM DTT) by using a 30-kDa-cutoff filter (6 ml, Merck Millipore) and *Mt*Fsr was concentrated to 900 µl. The concentrated sample was passed onto a Superdex 200 Increase 10/300 GL (GE Healthcare), equilibrated in storage buffer. *Mt*Fsr eluted at a flow rate 0.4 ml min^−1^ in a sharp Gaussian peak at an elution volume of 10.01 ml (Extended Data Fig. 2g). To determine the apparent molecular weight of *Mt*Fsr, standard proteins (conalbumin, aldolase and ferritin, purchased from GE Healthcare) were passed at the same flow rate and in the same buffer. The fractions of interest containing *Mt*Fsr were concentrated with a 30-kDa-cutoff centrifugal concentrator to 1 ml and the protein was directly used for enzymatic activity assays. The concentration of purified *Mt*Fsr, estimated by the Bradford method, was 3.41 mg ml^−1^.

### Purification of *Mt*Fsr for titrations and EPR spectroscopy

For the titrations and EPR spectroscopic measurements two separate purifications were carried out starting either with 34 g cells (wet weight) derived from a SO_3_^2−^-grown fermenter, or with 49.5 g cells (wet weight) derived from a SO_4_^2−^-grown fermenter. Cells were thawed under warm water and transferred to an anaerobic tent containing an atmosphere of N_2_/CO_2_ (90:10). Cells were lysed by osmotic shock through the addition of 180 ml and 240 ml lysis buffer (50 mM Tricine/NaOH pH 8.0, 2 mM DTT), respectively. The cell lysates were homogenized by sonication: 4 cycles at 72% intensity with 60 pulses followed by 1.30 minute break (probe MS76, SONOPULS Bandelin) and the cell debris was removed anaerobically via centrifugation (21,000*g*, 1 h at 10 °C). The supernatant was transferred in a Coy tent (N_2_/H_2_ atmosphere of 97:3), with yellow light at 20 °C.

The purification steps were carried out as described in ‘Purification of MtFsr for crystallization’. In the final purification step the buffer was exchanged by dilution and concentration in storage buffer (25 mM Tris–HCl pH 7.6, containing 10% v/v glycerol and 2 mM DTT) by using 30-kDa-cutoff filter (6 ml, Merck Millipore). *Mt*Fsr derived from the SO_3_^2−^-grown fermenter was concentrated to 18 mg ml^−1^ in a volume of 4.54 ml, and for the SO_4_^2−^-grown fermenter *Mt*Fsr was concentrated to 20 mg ml^−1^ in a volume of 1.24 ml. The protein concentrations were estimated by the Bradford method.

### Mass spectrometry identification

Purified *Mt*Fsr (1 μg) was digested with trypsin and analyzed by mass spectrometry (ThermoFisher Q Exactive HF coupled to an Easy-nLC 1200) as described in ref. 44.

### Protein crystallization

The purified enzymes were kept in 25 mM Tris–HCl pH 7.6, 10% v/v glycerol and 2 mM DTT. Fresh, unfrozen samples were immediately used for crystallization. Crystals were obtained anaerobically (N_2_/H_2_, 97:3) by initial screening at 20 °C using the sitting-drop method on 96-well MRC two-drop crystallization plates in polystyrene (SWISSCI) containing 90 µl of crystallization solution in the reservoir.

### Crystallization of *Mj*Fsr

*Mj*Fsr (0.5 µl) at a concentration of 6.1 mg ml^−1^ was mixed with 0.5 µl reservoir solution. Black, long, plate-shaped crystals appeared after a few days in the following crystallization conditions: 45% v/v 2-methyl-2,4-pentanediol, 100 mM Bis–Tris pH 5.5 and 200 mM calcium chloride.

### Crystallization of *Mt*Fsr

*Mt*Fsr at a concentration of 11 mg ml^−1^ was cocrystallized with FAD (0.5 mM final concentration) and F_420_H_2_ (15.5 µM final concentration). The protein sample (0.6 µl) was mixed with 0.6 µl reservoir solution. Thick, square-shaped, brown crystals appeared after a few days. The reservoir solution contained 200 mM lithium sulfate, 100 mM Bis–Tris, pH 5.5 and 25% w/v polyethylene glycol 3350.

### X-ray crystallography and structural analysis

Crystal handling was done inside the Coy tent under anaerobic atmosphere (N_2_/H_2_, 97:3). *Mj*Fsr crystals were directly plunged in liquid nitrogen, whereas *Mt*Fsr crystals were soaked in their crystallization solution supplemented with 20% v/v ethylene glycol as a cryo-protectant before being frozen in liquid nitrogen. Crystals were tested and collected at 100 K at the Synchrotron Source Optimisée de Lumière d’Énergie Intermédiaire du LURE (SOLEIL), PROXIMA-1 beamline; the Swiss Light Source, X06DA–PXIII; and at PETRA III, P11.

### *Mj*Fsr

After an X-ray fluorescence spectrum on the Fe K-edge, datasets were collected at 1.74013 Å to perform the single-wavelength anomalous dispersion experiment. Native datasets were collected at a wavelength of 0.97857 Å on the same crystal. Data were processed and scaled with autoPROC^45^. The resolution limits in each cell direction were as follows: *a* = 2.43 Å, *b* = 2.62 Å and *c* = 2.19 Å. Phasing (obtained maximum CFOM for the substructure determination was 69), density modification and automatic building were performed with CRANK-2 (ref. 46). The asymmetric unit of *Mj*Fsr contains two half homotetramers. The model was then manually built with Coot and further refined with PHENIX^47,48^. X-ray crystallographic data were twinned, and the refinement was performed by applying the following twin law -k, -h, -l. During the refinement translational-liberation screw was applied.

### *Mt*Fsr

Data were processed and scaled with autoPROC. The resolution limits in each cell direction were as follows: *a* = 1.69 Å, *b* = 1.55 Å and *c* = 1.81 Å. The structure was solved by molecular replacement with phaser from PHENIX, using *Mj*Fsr as a template^48^. The asymmetric unit of *Mt*Fsr contains four homotetramers. This crystalline form presents a notable translational noncrystallographic symmetry (14%). The model was then manually rebuilt with Coot and further refined with PHENIX. During the refinement, noncrystallographic symmetry and translational-liberation screw were applied. In the last refinement cycles, hydrogens were added in riding positions. Hydrogens were omitted from the final deposited model. In one of the chains (chain N), the lid region 204–253 has two different conformations, and both were tentatively modeled.

All models were validated through the MolProbity server (http://molprobity.biochem.duke.edu)^49^. B-factors, MolProbity scores and rotamer outliers in Extended Data Table 1 were calculated based on the available PDB structures with PHENIX. The other values in Extended Data Table 1 were derived from the original first PDB reports. Data collection and refinement statistics, as well as PDB identification codes for the deposited models and structure factors, are listed in Extended Data Table 1. Figures were generated with PyMOL (Schrödinger, LLC). Structural comparison was performed with the dissimilatory sulfite reductases from *D. vulgaris* (2V4J), *A. fulgidus* (3MM5) and with the assimilatory sulfite reductase from *E. coli* (1AOP) and *Z. mays* (5H92).

### Purification of the F_420_-reducing hydrogenase from *M. thermolithotrophicus*

*Mt*Frh was required to reduce F_420_ and was purified from the same batch of cells as *Mt*Fsr used for crystallization. The activity of *Mt*Frh after each purification step was followed by the reduction of methyl viologen in the N_2_/H_2_ tent (97:3). The assay was performed in 120 µl of 0.5 M KH_2_PO_4_/NaOH pH 7.6 containing 1.7 mM of oxidized methyl viologen. The addition of 2 µl from the fractions containing Frh led to a blue coloration.

*Mt*Frh was in the same pool as *Mt*Fsr used for crystallization, for the DEAE and the Q Sepharose columns. The Q Sepharose column performed the separation of the two target proteins. *Mt*Frh eluted between 0.48 and 0.49 M NaCl from the Q Sepharose column. The filtered sample was applied to a 10-ml hydroxyapatite type 1 (Bio-Scale Mini CHT cartridges, BioRad) equilibrated with HAP buffer (20 mM K_2_HPO_4_/HCl pH 7.0 and 2 mM DTT). The column was then washed with 2 CV of HAP buffer. The elution was performed with a gradient of 0.02 to 0.5 M K_2_HPO_4_ in 60 min at a flow rate of 2 ml min^−1^ with 3-ml fractions. *Mt*Frh eluted between 0.22 and 0.37 M K_2_HPO_4_ and the respective fractions were pooled. The pool was diluted 1:1 with the HIC buffer (25 mM Tris–HCl pH 7.6, 2 M (NH_4_)_2_SO_4_ and 2 mM DTT). The filtered sample was applied onto a Source15Phe 4.6/100 PE column (GE Healthcare) previously equilibrated with the HIC buffer. The column was then washed with 2 CV of 25 mM Tris–HCl pH 7.6, 1.0 M (NH_4_)_2_SO_4_ and 2 mM DTT buffer. *Mt*Frh was eluted in a gradient of 1 to 0 M (NH_4_)_2_SO_4_ in 30 min at a flow rate of 0.8 ml min^−1^ and a fractionation size of 1 ml. *Mt*Frh eluted between 0.4 and 0.15 M (NH_4_)_2_SO_4_ and the respective fractions were pooled. The buffer was exchanged for the storage buffer (25 mM Tris–HCl pH 7.6, containing 10% v/v glycerol and 2 mM DTT) by using a 30-kDa-cutoff filter (6 ml, Merck Millipore) and *Mt*Frh was concentrated to 4.97 mg ml^−1^ in 100 µl. The purified sample was aliquoted and anaerobically flash frozen in liquid N_2_ and stored at −80 °C. *Mt*Frh lost its activity after more than one cycle of thawing-freezing.

### Purification of oxidized F_420_

Since F_420_ is highly sensitive to light, all steps were carried out under yellow light or by covering the sample with aluminum foil. About 10 g (wet weight) of *M. thermolithotrophicus* cells from a 1.5-l fermenter were anaerobically lysed by osmotic shock and sonication (see above). The sample was centrifuged at 45,000*g* for 60 min at 4 °C. The supernatant was transferred in a Coy tent containing an atmosphere of N_2_/H_2_ (97:3). The sample was filtered and passed onto a 30-ml DEAE Sepharose column equilibrated with 50 mM Tricine/NaOH pH 8.0 and 2 mM DTT. F_420_ was eluted by a gradient of 0 to 0.6 M NaCl. The samples containing F_420_ were determined on the basis of the absorbance profile at 420 nm and eluted between 0.48 M and 0.58 M NaCl. Pooled fractions were moved outside the tent and diluted with one volume of HIC-F_420_ buffer (25 mM Tris HCl pH 7.6, 2 M (NH_4_)_2_SO_4_). (NH_4_)_2_SO_4_ powder was directly added to the diluted sample to reach a final concentration of 3 M (NH_4_)_2_SO_4_ and was stirred for one hour at room temperature. The sample was centrifuged at 4,000*g* for 20 minutes at room temperature. The supernatant was filtered through a 0.2-µm filter and loaded on a 30-ml Phenyl-Sepharose high-performance column, equilibrated with HIC-F_420_ buffer. F_420_ was eluted by washing the column with the HIC-F_420_ buffer, at a flow rate of 2 ml min^−1^ and 1-ml fractions were collected. The fractions containing F_420_ were pooled and filtered through a 0.2-µm filter. The sample was diluted by 50 volumes of 5 mM Tris–HCl pH 8.0 and loaded overnight on a 5-ml Q Sepharose high-performance column, equilibrated in 5 mM Tris–HCl pH 8.0. The following steps were performed at 4 °C. The column containing the bound F_420_ was washed with 5 CV of 20 mM (NH_4_)HCO_3_ precooled at 4 °C. F_420_ elution was performed by adding 1 M (NH_4_)HCO_3_ and collected in a brown serum flask. (NH_4_)HCO_3_ was removed by evacuation at 37 °C for 2 hours under constant stirring. (NH_4_)HCO_3_-free F_420_ powder was obtained by freeze drying. The purity of the preparation was checked by measuring the ratio of Abs_247_/Abs_420_ in 25 mM Tris buffer pH 8.8. A pure sample would have a ratio value of 0.85 (ref. 50). F_420_ concentration was estimated by measuring the absorbance at 420 nm in 25 mM Tris buffer pH 7.5 (*ε*_420_ = 41.4 mM^−1^ cm^−1^). The final concentration of oxidized F_420_ used for this study was 3.15 mM and 7.53 mM.

### Reduction of F_420_ for enzyme assays

For enzyme activity assays and cocrystallization of *Mt*Fsr with F_420_H_2_, the oxidized F_420_ needed to be reduced. Dithionite was not used since it contains 10–20% (m/m) sodium sulfite and generates further SO_3_^2−^ as product. All steps were performed under the strict exclusion of oxygen and under yellow light. First, the aerobic gas phase of the F_420_ stock was exchanged several times for N_2_. The sample was then transferred in a Coy tent with an atmosphere containing a N_2_/H_2_ mixture (97:3). The reduction took place in 1.4 ml 200 mM KH_2_PO_4_, pH 7.0, 0.5 mM F_420_, and 5 µl of 5 mg ml^−1^ purified *Mt*Frh was added. Outside the tent, in a brown serum flask, the gas phase was exchanged three times for H_2_ and CO_2_ by evacuation and gassing with 1 × 10^5^ Pa H_2_ and CO_2_ (80:20) at room temperature. The reduction of F_420_ was observed by the color shift from yellow to transparent. Frh was removed by passing the sample through a 10-kDa-cutoff filter. Since reduced F_420_ is not stable and oxidizes with time, aliquoted F_420_H_2_ without Frh was immediately flash frozen in liquid N_2_ and stored at −80 °C.

### Reduction of F_420_ for redox titrations

F_420_ is the physiological electron donor for Fsr and was therefore used as the reductant for the redox titrations. Oxidized F_420_ was purified as described before. Since both the reduction of F_420_ with Frh is not complete and F_420_H_2_ is not stable over time, we reduced F_420_ with sodium borohydride, as previously described^51^. The reduction of F_420_ was performed in an anaerobic chamber with an N_2_/H_2_ atmosphere of 97:3 at 25 °C. F_420_H_2_ was generated by reducing 100 µl F_420_ at 7.53 mM with a few sodium borohydride crystals in a 10 mM Tris-HCl solution at pH 7.6, followed by destruction of excess borohydride by acidification with 50 µl 1 M hydrochloric acid. After the hydrogen evolution ceased, the pH was readjusted by the addition of 50 µl 1 M Tris–HCl pH 8.0. The generated F_420_H_2_ was prepared freshly for each experiment and used immediately.

### Enzymatic assays

Enzymatic Fsr measurements were performed in 200 mM KH_2_PO_4_ buffer pH 7.0 under strict exclusion of hydrogen and oxygen. F_420_ was reduced by Frh as previously described. The oxidation of the reduced electron donor F_420_ was followed spectrophotometrically at 420 nm. For F_420_H_2_, a molecular extinction coefficient of 33.82 mM^−1^ cm^−1^ at 420 nm was experimentally determined for the above-mentioned conditions.

The assays for the specific enzyme activity were performed at 65 °C in a 1-ml quartz cuvette closed with a butyl rubber stopper. The gas phase of the cuvette was exchanged several times with N_2_. To monitor the reduction of SO_3_^2−^, 1.4 mM Na_2_SO_3_ and 47.3 µM F_420_H_2_ were added to the KH_2_PO_4_ buffer. Once the spectrophotometer (Agilent Cary 60 UV–Vis) displayed a stable signal, the reaction was started by the addition of 0.19 µg *Mt*Fsr. To investigate whether *Mt*Fsr can use substrates other than SO_3_^2−^, we provided 1.4 mM of disodium thiosulfate (S_2_O_3_^2−^), 1.4 mM sodium nitrite (NO_2_^−^) or 1.4 mM disodium selenite (SeO_3_^2−^). We further tested whether *Mt*Fsr can function in the reverse way by providing 1.4 mM Na_2_S as an electron donor and 47.3 µM of oxidized F_420_. All experiments were performed in triplicate.

The app*K*_m_ and app*V*_max_ of *Mt*Fsr for SO_3_^2−^ and NO_2_^−^ were determined at 50 °C under an anaerobic atmosphere (100% N_2_). The assays were performed in 96-deep-well plates and monitored spectrophotometrically (FLUOstar Omega Multi-Mode Microplate Reader). To determine the app*K*_m_ and app*V*_max_ of *Mt*Fsr, 0–500 µM Na_2_SO_3_ or NaNO_2_ and 50 µM F_420_H_2_ were added to the 200 mM KH_2_PO_4_ buffer pH 7.0 and the reaction was started by the addition of 3.8 ng *Mt*Fsr. All experiments were performed in triplicate with a standard deviation represented by the ± sign. Kinetic parameters were calculated based on the ic50.tk server by applying a Hill coefficient of 1 (http://www.ic50.tk/kmvmax.html).

### EPR spectroscopy

The midpoint potentials of the [4Fe‒4S] centers and the siroheme of *Mt*Fsr were determined from EPR signal intensities and EPR integrals of the various redox states. All titrations were performed in a Coy tent (N_2_/H_2_, 97:3), at 25 °C in the dark. A volume of 3.32 or 3 ml for the reductive or oxidative titrations with F_420_H_2_ or potassium ferricyanide at an initial *Mt*Fsr concentration of 4.07 or 2.7 mg ml^−1^ (in 100 mM Tris–HCl, pH 7.6), respectively, was stirred under anaerobic conditions. The solution potential was measured with an InLab ARGENTHAL (Mettler) microelectrode (Ag/AgCl, +207 mV versus H_2_/H^+^ with in-built platinum counter electrode) in the presence of the respective mediator mix. *Mt*Fsr was preincubated for 30 minutes before each titration with the mediator mix and assay buffer. The amount of *Mt*Fsr available and the necessary protein concentration to obtain a satisfying signal-to-noise ratio for the EPR spectra precluded multiple titrations. Thus, values reported were from a single redox titration for the siroheme and from two redox titrations for the Fe/S signals.

The mediator mix for the reductive titration contained methylene blue, resorufin, indigo carmine, 2-hydroxy-1,4-naphthoquinone (50 µM), sodium anthraquinone-2-sulfonate, phenosafranin, safranin T, neutral red, benzyl and methyl viologen (all at a final concentration of 25 µM, except 2-hydroxy-1,4-naphthoquinone). For the oxidative titration the mediator mix contained methylene blue, resorufin, indigo carmine, 2-hydroxy-1,4-naphthoquinone (all at a final concentration of 20 µM). After adjustment of the potential by microliter additions of F_420_H_2_ or potassium ferricyanide and 3 minutes equilibration, EPR samples were taken. For this, 300 µl of the mix were withdrawn, removed from the anaerobic glovebox in EPR tubes after attachment of a 5-cm piece of 3 mm × 7 mm (internal diameter × outer diameter) natural rubber tubing sealed with a 5-mm outer diameter acrylic glass stick at the other end. The samples were stored in liquid nitrogen until EPR spectra were recorded.

*Mt*Fsr as isolated was already in a partially reduced state. To obtain the completely oxidized form, 675 µl Fsr at 20 mg ml^−1^ was incubated for 30 minutes with 2 mM methylene blue. The sample was then passed through a Sephadex G-25M column (previously equilibrated with 100 mM Tris–HCl pH 7.6) to remove the methylene blue. This methylene blue-treated Fsr (1.28 ml) was collected at a concentration of 5.65 mg ml^−1^ and 300 µl was directly taken frozen for EPR spectroscopy of Fsr in its oxidized form.

Samples from the same methylene blue-treated Fsr (passed through a Sephadex G-25M column) at 5.09 mg ml^−1^ final concentration were incubated for 5 minutes with 10 mM Na_2_SO_3_, and then stored in liquid nitrogen.

All EPR spectra were recorded on a Bruker Elexsys E580 X band spectrometer (digitally upgraded) with a 4122HQE cavity linked to an ESR 900 Oxford Instruments helium flow cryostat. Cryocooling was performed by a Stinger (Cold Edge Technologies) closed-cycle cryostat driven by an F-70 Sumitomo helium compressor. Our local glassblower produced EPR tubes from Ilmasil PN tubing (outer diameter 4.7 mm and 0.5 mm wall thickness, Qsil). Before use, the tubes were extensively cleaned with pipe cleaners to remove inadvertent contaminants. EPR spectra were simulated with Easyspin^52^. The concentration of Fsr for the spin integration (using a 1 mM Cu^2+^–EDTA solution as standard) was obtained by dividing the Fe concentration, as determined with the ferene method^29^, by 24, since siroheme does not release Fe. Fitting to the Nernst equation was performed in Excel.

### High-resolution clear-native PAGE

To visualize the expression levels of Fsr in HS^−^- versus SO_3_^2−^-grown cultures, and to estimate the oligomerization of Fsr, high-resolution clear-native–PAGE (hrCN–PAGE) was performed. 10 ml of *M. thermolithotrophicus* and *M. jannaschii* cultures, with either 2 mM Na_2_S or 2 mM Na_2_SO_3_ as sulfur source, were grown for one night at 65 °C, with standing. Cells were collected by anaerobic centrifugation at 6,000*g* for 20 min at room temperature and the cell pellets were resuspended in 2 ml of 50 mM Tricine/NaOH pH 8.0 and 2 mM DTT. The cells were anaerobically sonicated four times at 70% intensity for 10 seconds, followed by a 30-second break (MS 73 probe, SONOPULS Bandelin). The hrCN–PAGE was run anaerobically and the protocol was adapted from ref. 53. Linear polyacrylamide gradient gels (8–15%) were prepared under aerobic conditions but then transferred into an anoxic chamber (atmosphere of N_2_/CO_2_, 90:10), where the gels were equilibrated in anaerobic cathode buffer (50 mM Tricine; 15 mM Bis–Tris, pH 7.0; 0.05% w/v sodium deoxycholate; 0.01% w/v dodecyl maltoside and 2 mM DTT) overnight. Fresh and anaerobic samples were diluted with the lysis buffer to a final concentration of 1 mg ml^−1^ and a volume of 12 µl per sample was loaded onto the gel, as well as 2 µl of the NativeMark Unstained Protein Standard ladder (ThermoFisher). Glycerol (20% v/v final) was added to each sample and 0.001% w/v Ponceau S served as a marker for protein migration. The electrophoresis anode buffer contained 50 mM Bis–Tris buffer pH 7.0 and 2 mM DTT. The hrCN gels were run with a constant 40-mA current (PowerPac Basic Power Supply, BioRad). After electrophoresis, the protein bands were aerobically stained with Instant Blue (Expedeon).

### Reporting summary

Further information on research design is available in the Nature Portfolio Reporting Summary linked to this article.

## Online content

Any methods, additional references, Nature Portfolio reporting summaries, source data, extended data, supplementary information, acknowledgements, peer review information; details of author contributions and competing interests; and statements of data and code availability are available at 10.1038/s41589-022-01232-y.

### Supplementary information

Supplementary Information Supplementary Figs. 1–11 and Table 1.

Reporting Summary

### Source data

Source Data Table 1 Statistical source data for the nitrite/sulfite *K*_m_ measurements for *Mt*Fsr.

Source Data Extended Data Fig. 2 Unprocessed gels for Extended Data Fig. 2.

Source Data Extended Data Fig. 2 Statistical source data for Extended Data Fig. 2.

Source Data Extended Data Fig. 4 Statistical source data for Fig. 4c.

## Figures and Tables

**Extended Data Table 1:**
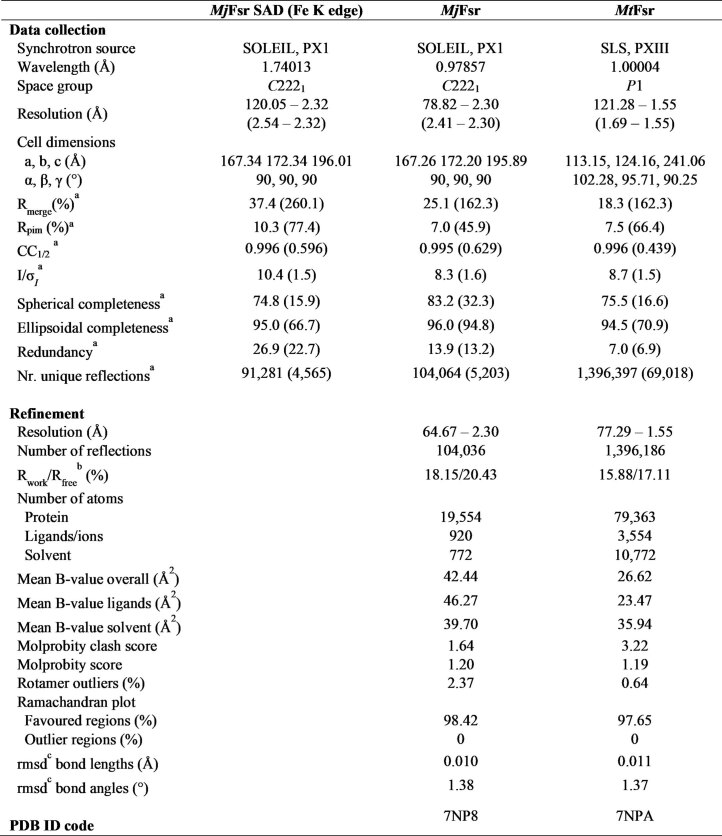
X-ray analysis statistics for Fsr

^a^Values relative to the highest resolution shell are within parentheses.

^b^ R_free_ was calculated as the R_work_ for 5% of the reflections that were not included in the refinement.

^c^ rmsd, root mean square deviation.

## Data Availability

The crystal structures have been deposited in the Protein Data Bank under accession codes: 7NP8 for *Mj*Fsr and 7NPA for *Mt*Fsr. Raw crystallographic data have been deposited on Zenodo: 10.5281/zenodo.4751125. The data for this study are available within the paper and its Supplementary Information. Source data are provided with this paper.
