# Supplementary material for: Structures of the sulfite detoxifying F420-dependent enzyme from Methanococcales
Source: Nat Chem Biol. 2023 Jan 19;19(6):695–702. doi: 10.1038/s41589-022-01232-y (PMC10229431; doi:10.1038/s41589-022-01232-y)
Supplement: Supplementary file 1 — Supplementary Figs. 1–11 and Table 1. [file 41589_2022_1232_MOESM1_ESM.pdf]

nature chemical biology

Article

https://doi.org/10.1038/s41589-022-01232-y

Structures of the sulfite detoxifying F_{420}-dependent enzyme from *Methanococcales*

In the format provided by the authors and unedited

Table of contents

1. Supplementary Fig. 1 Page 2
2. Supplementary Fig. 2 Page 3-4
3. Supplementary Fig. 3 Page 5
4. Supplementary Fig. 4 Page 6
5. Supplementary Fig. 5 Page 7
6. Supplementary Fig. 6 Page 8
7. Supplementary Fig. 7 Page 9
8. Supplementary Fig. 8 Page 10
9. Supplementary Fig. 9 Page 11
10. Supplementary Fig. 10 Page 12
11. Supplementary Fig. 11 Page 13
12. Supplementary Table 1 Page 14-15

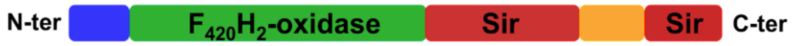

F_{420}-oxidoreductases
F_{420}-oxidase domain of Fsr Group I and II[1]
F_{420}-reducing [NiFe(Se)]-hydrogenases (FrhB)[2]
$F_{420}H_2$:quinone oxidoreductase (FqoF)[3]
$F_{420}H_2$:phenazine oxidoreductase (FpoF)[4]
F_{420}-dependent glutamate synthase (GOGAT)[5]
Formate dehydrogenase (FdhB)[6]

Sulfite reductases	Biological function
Group I Fsr[1]	detoxification/assimilatory
Group II Fsr[7]	unknown
DsrA[8,9]	dissimilatory (inactive)
DsrB[8,9]	dissimilatory
ArsC[1]	dissimilatory
alSir/Group I Dsr-LP[5]	assimilatory
Group III Dsr-LP[5]	unknown
aSir[10,11]	assimilatory

Supplementary Fig. 1. Domain affiliations of the F_{420}-dependent sulfite reductase (Fsr). Top panel, Fsr domain organization. Its N-terminal half belongs to the F_{420}-oxidoreductases (left table) as the F_{420}-reducing hydrogenase β-subunit (FrhB), together with the $F_{420}H_2$:quinone oxidoreductase (FqoF), the $F_{420}H_2$:phenazine oxidoreductase (FpoF), the putative F_{420}-dependent glutamate synthase and formate dehydrogenase (GOGAT and FdhB). Its C-terminal half corresponds to the sulfite reductases (right table). Sulfite reductases are generally classified into assimilatory or dissimilatory. Group I Fsr: Group I F_{420}-dependent sulfite reductase, Group II Fsr: Group II F_{420}-dependent sulfite reductase, DsrA: dissimilatory sulfite reductase α-Subunit, DsrB: dissimilatory sulfite reductase β-Subunit, ArsC: anaerobic sulfite reductase subunit C, alSir/Group I Dsr-LP: assimilatory-type low-molecular-weight sulfite reductase/Group I dissimilatory sulfite reductase-like proteins, Group III Dsr-LP: Group III dissimilatory sulfite reductase-like proteins, aSir: assimilatory sulfite reductase. References[1-11] are cited accordingly.

a

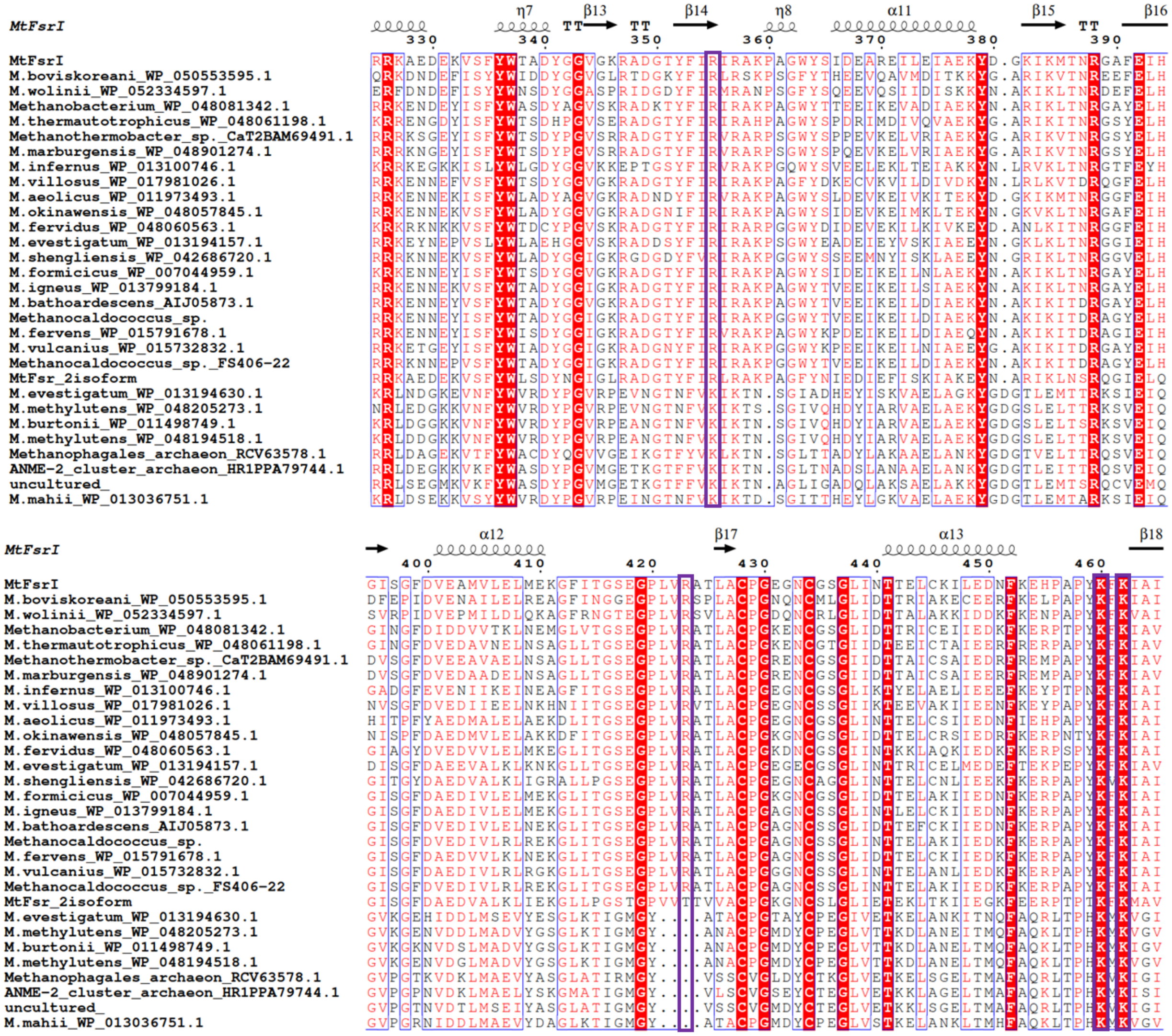
MtFsrI
η7
β13
TT
β14
η8
α11
β15
β16
330
340
350
360
370
380
390
MtFsrI
M.boviskoreani_WP_050553595.1
M.wolinii_WP_052334597.1
Methanobacterium_WP_048081342.1
M.thermautotrophicus_WP_048061198.1
Methanothermobacter_sp._CaT2BAM69491.1
M.marburgensis_WP_048901274.1
M.infernus_WP_013100746.1
M.villosus_WP_017981026.1
M.aeolicus_WP_011973493.1
M.okinawensis_WP_048057845.1
M.fervidus_WP_048060563.1
M.evestigatum_WP_013194157.1
M.shengliensis_WP_042686720.1
M.formicicus_WP_007044959.1
M.igneus_WP_013799184.1
M.bathoardescens_AIJ05873.1
Methanocaldococcus_sp.
M.fervens_WP_015791678.1
M.vulcanius_WP_015732832.1
Methanocaldococcus_sp._FS406-22
MtFsr_2isoform
M.evestigatum_WP_013194630.1
M.methylutens_WP_048205273.1
M.burtonii_WP_011498749.1
M.methylutens_WP_048194518.1
Methanophagales_archaeon_RCV63578.1
ANME-2_cluster_archaeon_HR1PPA79744.1
uncultured_
M.mahii_WP_013036751.1
MtFsrI
α12
β17
α13
β18
400
410
420
430
440
450
460

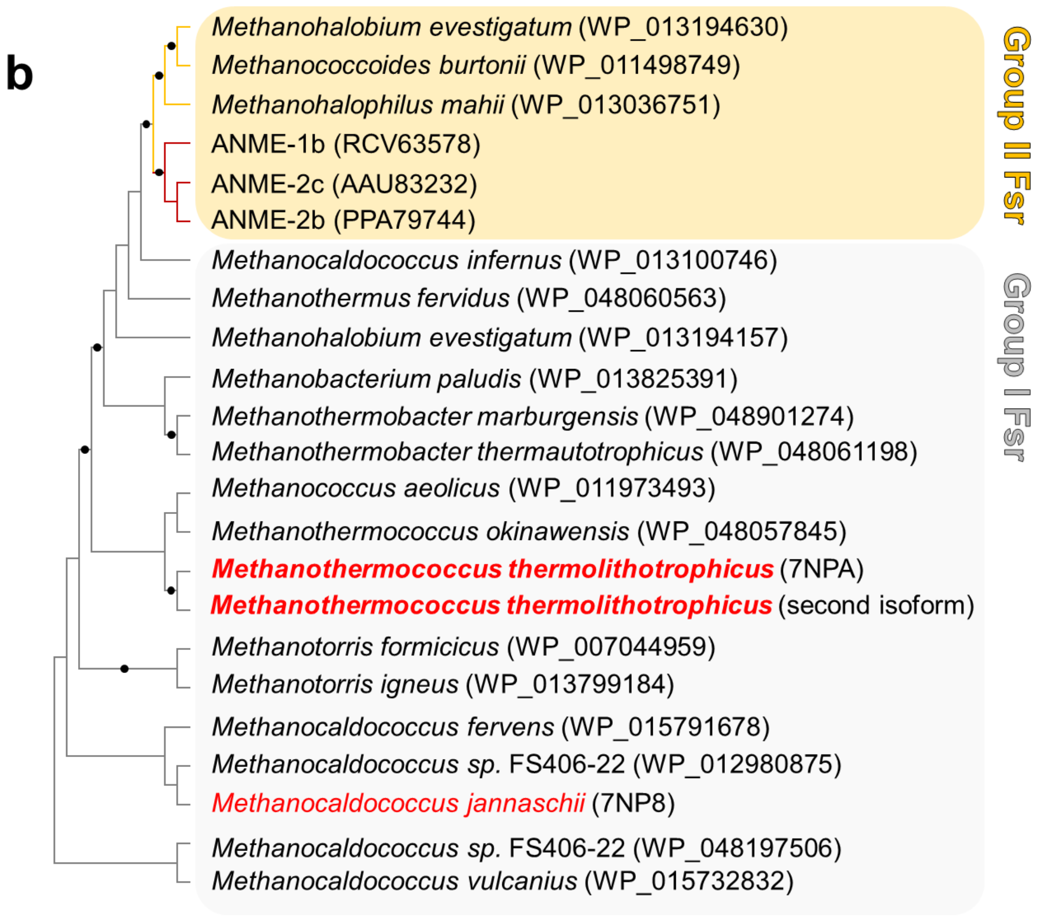

Supplementary Fig. 2. Differences between Group I and II Fsr. **a,** Sequence conservation across Group I and II Fsr on a selected stretch from the sulfite reductase domain. Perfectly conserved residues are highlighted with a red background. The purple squares highlight the four perfectly conserved residues across functional sulfite reductases (in *M. thermolithotrophicus*: Arg355, Arg423, Lys460 and Lys462). Sequence alignment was done using Clustal Omega[12], secondary structure prediction was performed with ESPript 3.0[13]. **b,** A neighbor-joining tree was constructed based on the phylogenetic analysis from Yu et al[7], including the two Fsr sequences from *M. thermolithotrophicus* (in bold red). Protein sequences were obtained from NCBI using BLASTP (E-value cut-off of 1e1), the sequences were then aligned using MUSCLE[14]. Phylogenetic analysis were performed using MEGA11[15]. Protein accession numbers from the NCBI database are shown in parentheses and black dots on the branches represent bootstrap values ≥ 90 %.

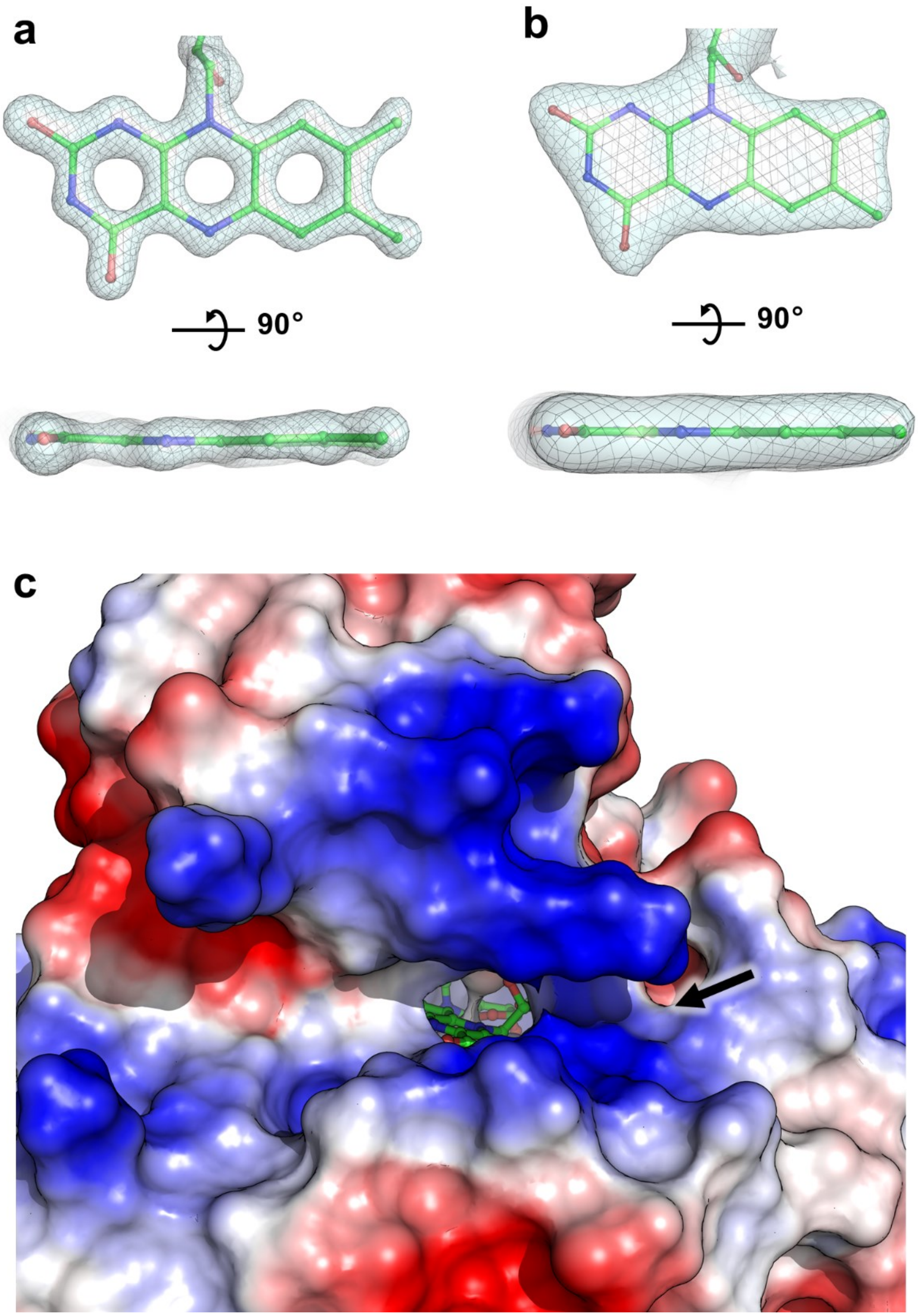

Supplementary Fig. 3. The Flavin conformation and F_{420} docking site. **a** and **b**, 2F_o-F_c map for the FAD in *Mt*Fsr and *Mj*Fsr contoured to 3- and 1.5-σ, respectively. The isoalloxazine heterocycle is only slightly bent. **c**, Electrostatic charge profile around the $F_{420}H_2$-oxidase active site in *Mt*Fsr. Acidic to basic patches on the surface are coloured in red and blue, respectively. The $F_{420}H_2$ is suspected to bind to the positively charged area (indicated by a black arrow) surrounding the FAD.

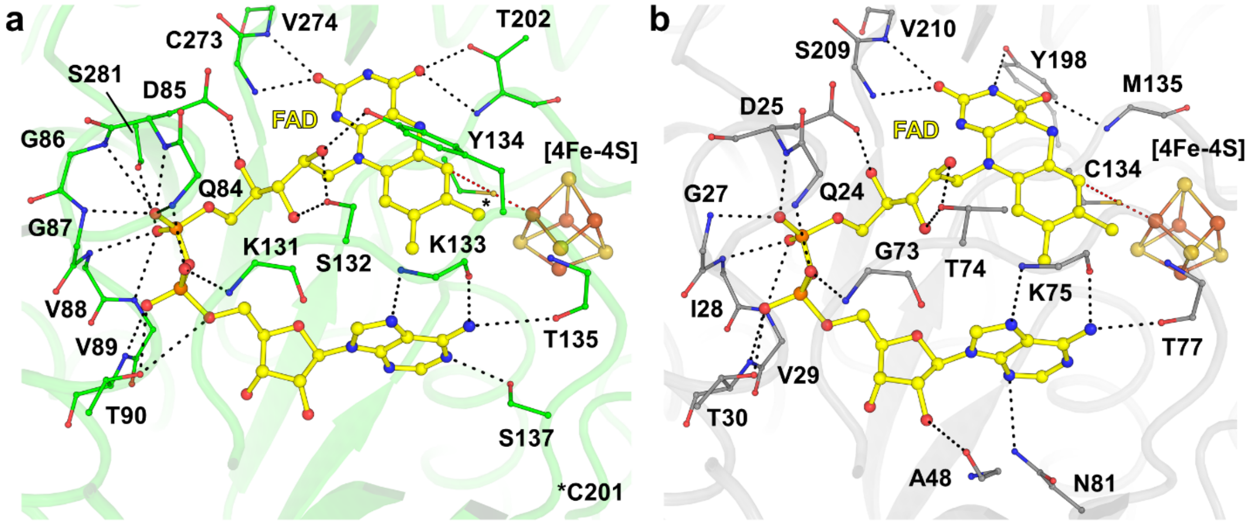

Supplementary Fig. 4. Comparison of the FAD binding site of the $F_{420}H_2$-oxidase domain from *Mt*Fsr and FrhB from *M. marburgensis.* **a**, Close-up of the FAD binding site in the $F_{420}H_2$-oxidase domain from *Mt*Fsr. **b**, FAD binding in FrhB from *M. marburgensis* (PDB 4OMF). The residues binding the FAD are represented in balls and sticks and hydrogen bonds involved in FAD binding are shown by black dashes. The connection between the [4Fe-4S]-cluster I and the isoalloxazine ring from the FAD, established by a cysteine, are shown by red dashes.

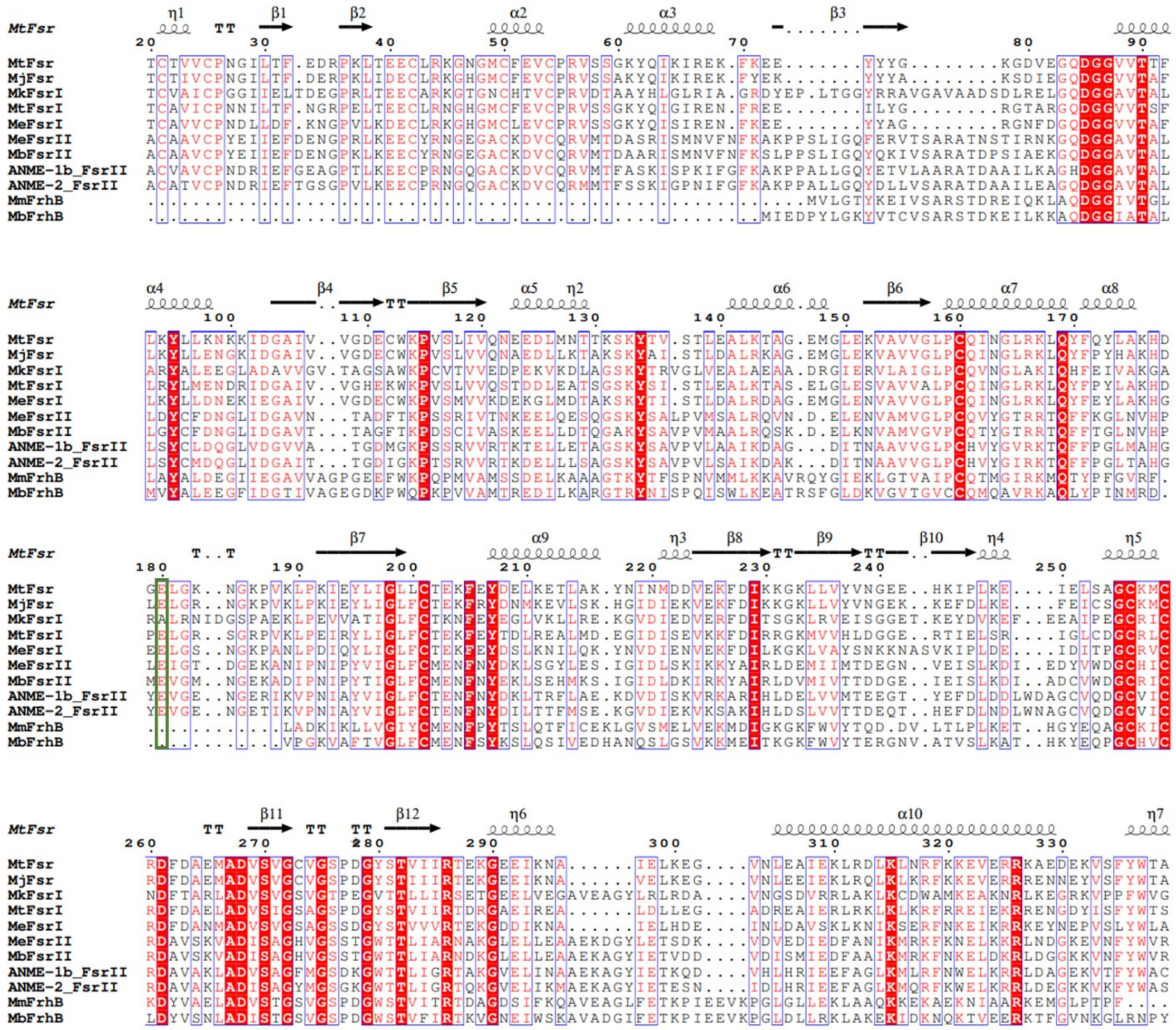

Supplementary Fig. 5. Sequence conservation across Group I and II Fsr as well as FrhB. Perfectly conserved residues are highlighted with a red background. The glutamate involved in the [4Fe-4S]-cluster 3 binding is shown by a green square. MtFsr: *M. thermolithotrophicus*, MjFsr: *M. jannaschii* (Q58280.1); MkFsrI: *Methanopyrus kandleri* (WP_011019168.1); MtFsrI: *Methanothermobacter marburgensis* (ADL58324.1); MeFsrI: *Methanohalobium evestigatum* (WP_013194157.1); MeFsrII: *Methanohalobium evestigatum* (WP_013194630.1); MbFsrII *Methanococcoides burtonii* (WP_011498749.1); ANME-1b_FsrII: Fsr from Methanophagales archaeon belonging to ANME-1 cluster (RCV63578.1); ANME-2_FsrII, Fsr from ANME-2 cluster archaeon HR1 (PPA79744.1); MmFrhB: *Methanothermobacter marburgensis* (ADL59254.1); MbFrhB *Methanosarcina barkeri* (WP_048177139.1). Sequence alignment was done using Clustal Omega[16], secondary structure prediction was performed with ESPript 3.0[13].

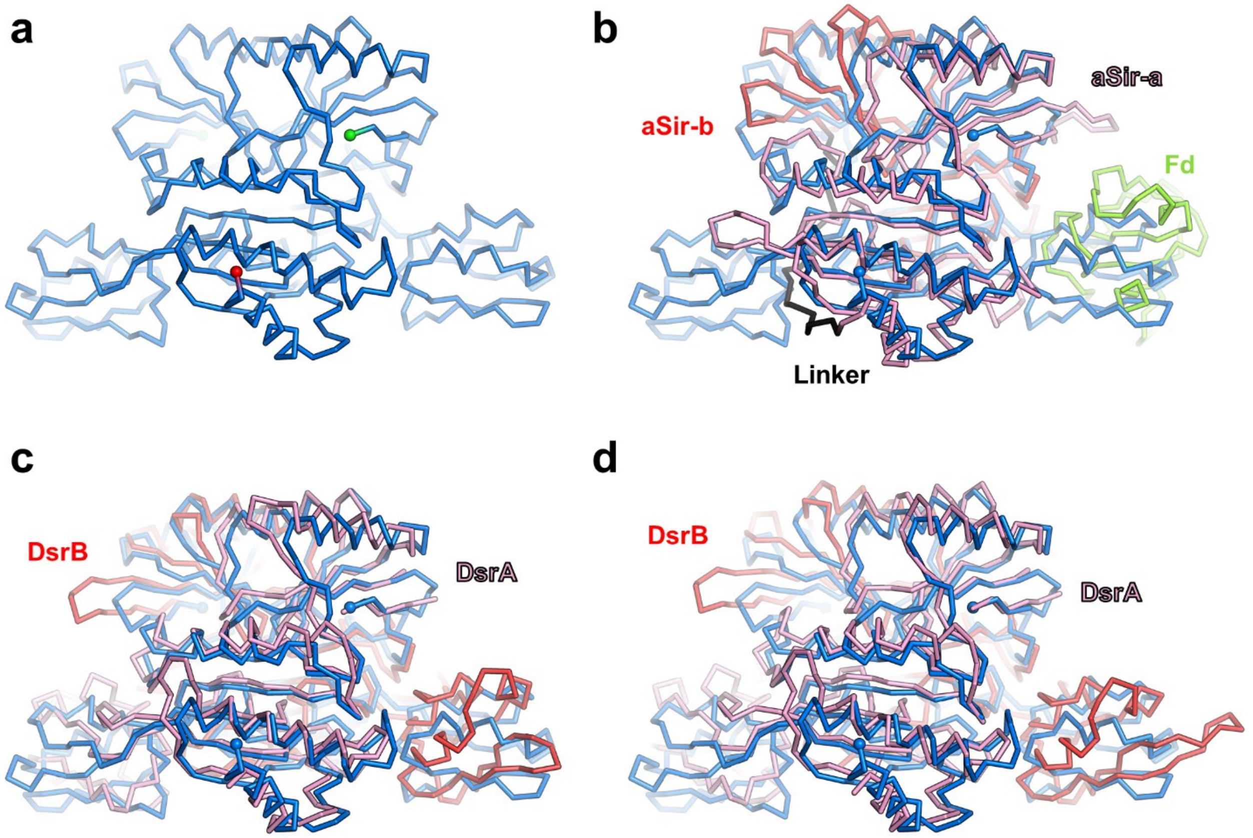

Supplementary Fig. 6. Fsr shares the common fold of sulfite reductases. **a**, The dimeric sulfite reductase domains of *Mt*Fsr with its inserted ferredoxin domains are represented in blue ribbon. N- and C-terminus of the Sir domain are shown in green and red spheres, respectively. **b**, Overall superposition of dimeric *Mt*Fsr (blue) with the aSir from *Zea mays* (PDB 5H92, whole chain rmsd=2.434 Å for 142-Cα aligned and a rmsd=0.964 Å for the most conserved region with 47-Cα aligned). The aSir-a part from *Zea mays* is coloured in light pink, and the aSir-b part is coloured in red. **c**, Overall superposition of dimeric *Mt*Fsr with DsrAB (DsrA in pink, DsrB in red) from *Archaeoglobus fulgidus* (PDB 3MM5, whole chain rmsd=4.152 Å for 225-Cα aligned and an rmsd=0.921 Å with 53-Cα aligned for the most conserved region on one *Mt*Fsr monomer). **d**, Overall superposition of dimeric *Mt*Fsr with DsrAB (DsrA in pink, DsrB in red) from *Desulfovibrio vulgaris* (PDB 2V4J, whole chain rmsd=2.819 Å for 157-Cα aligned and a rmsd=0.961 Å with 51-Cα aligned for the most conserved region on one *Mt*Fsr monomer). b-d, The extensions contained in aSir and DsrAB which are not common to Fsr have been removed for clarity.

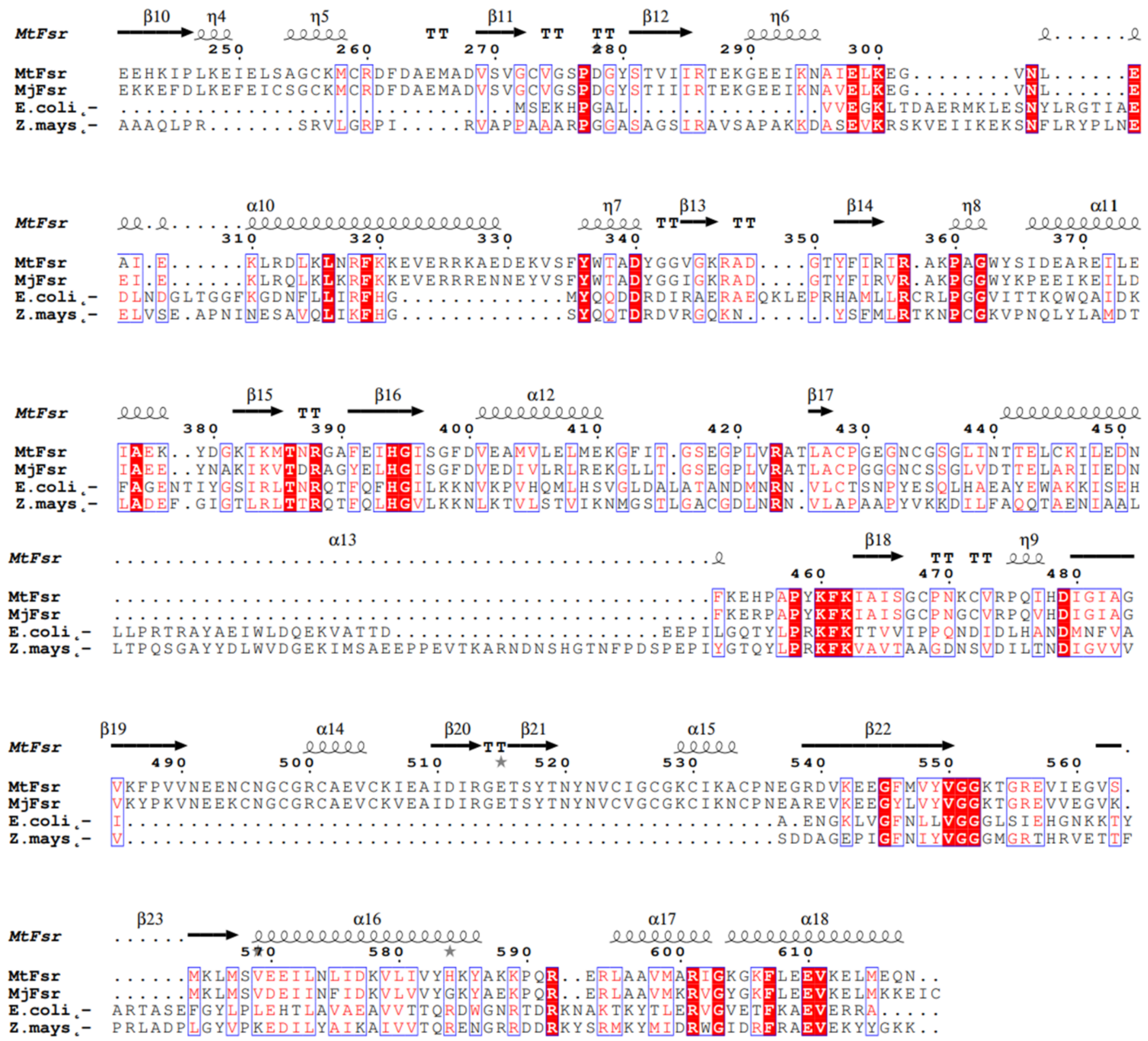

Supplementary Fig. 7. Sequence conservation across the C-terminal half of Fsr (*Mt*Fsr: 241-618) and aSir-a. Perfectly conserved residues are highlighted with a red background. MtFsr: *M. thermolithotrophicus*; MjFsr: *M. jannaschii*; Z.mays*: Zea mays* (PDB 5H92, for residues: 1-392); E.coli: *Escherichia coli* (PDB 2GEP, for residues: 1-327). Sequence alignment was done using Clustal Omega[16], secondary structure representation was performed with ESPript 3.0[13]. Arg355 seems not conserved in *E. coli* and *Z. mays* due to a shift of 2 residues in the alignment.

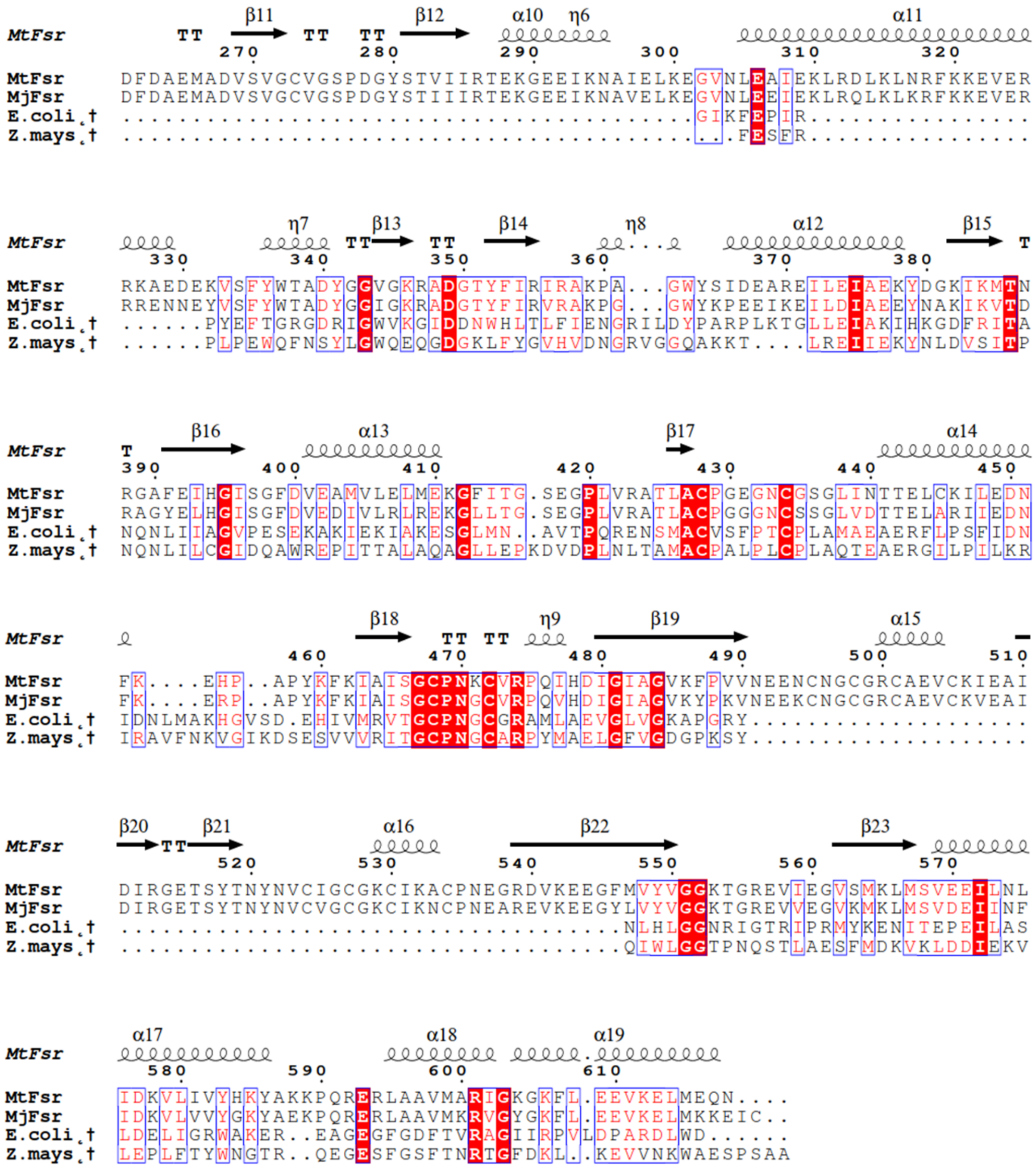

Supplementary Fig. 8. Sequence conservation across the C-terminal half of Fsr (*Mt*Fsr: 261-618) and aSir-b. Perfectly conserved residues are highlighted with a red background. MtFsr: *M. thermolithotrophicus*; MjFsr: *M. jannaschii*; Z. mays*: Zea mays* (PDB 5H92, used residues: 393-653); E.coli: *Escherichia coli* (PDB 2GEP, shown residues: 328-570). Sequence alignment was done using Clustal Omega[16], secondary structure representation was performed with ESPript 3.0[13].

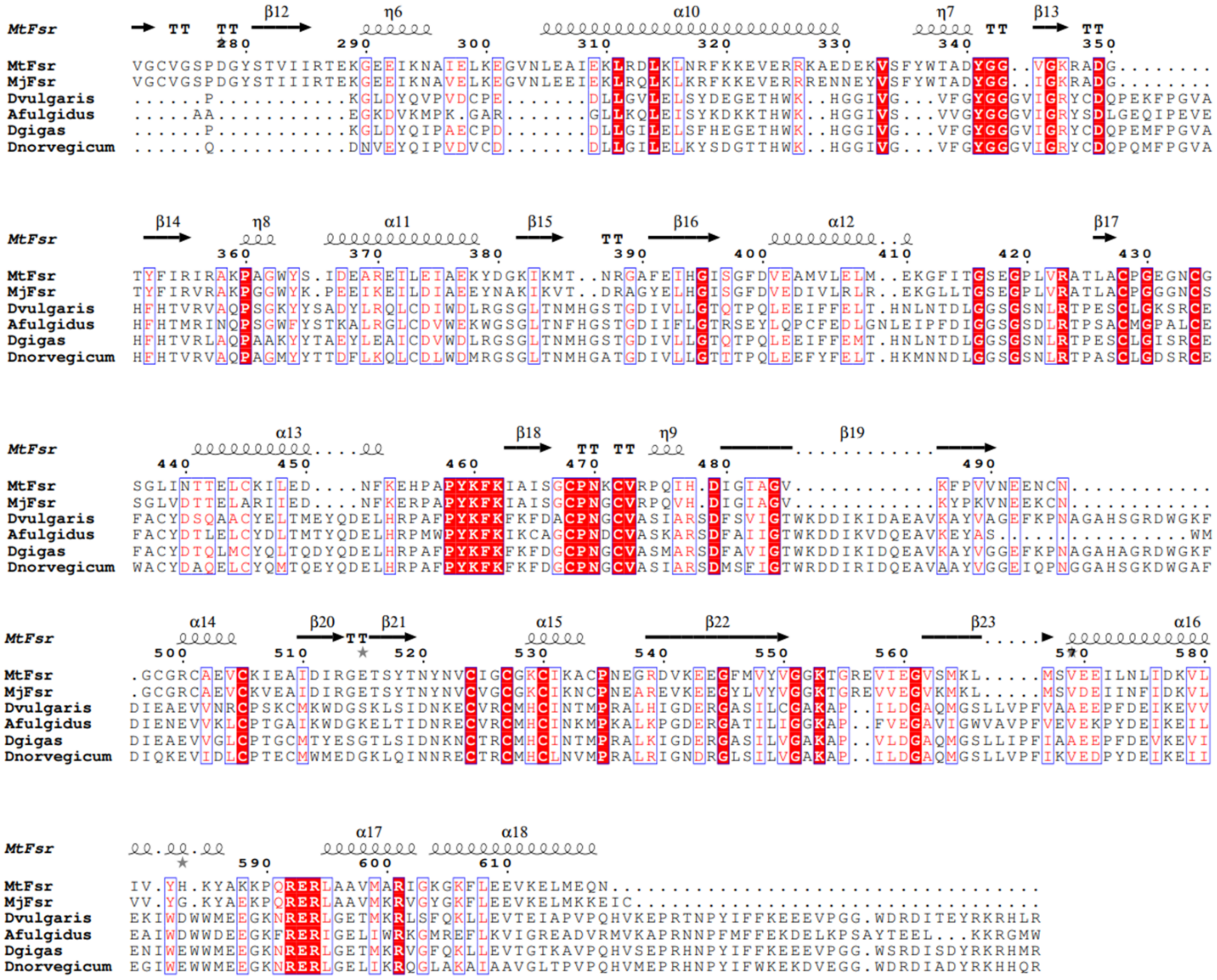

Supplementary Fig. 9. Sequence conservation across the C-terminal half of Fsr (*Mt*Fsr: 271-618) and DsrA. Perfectly conserved residues are highlighted with a red background. MtFsr: *M. thermolithotrophicus*; MjFsr: *M. jannaschii*. Dvulgaris*: D. vulgaris* (PDB 2V4J); Afulgidus: *Archaeoglobus fulgidus* (PDB 3MM5); Dgigas: *Desulfovibrio gigas* (PDB 3OR1); Dnorvegicum: *Desulfomicrobium norvegicum* (PDB 2XSJ). Sequence alignment was done using Clustal Omega[16], secondary structure representation was performed with ESPript 3.0[13]. Arg355 seems not conserved due to a shift of one residue.

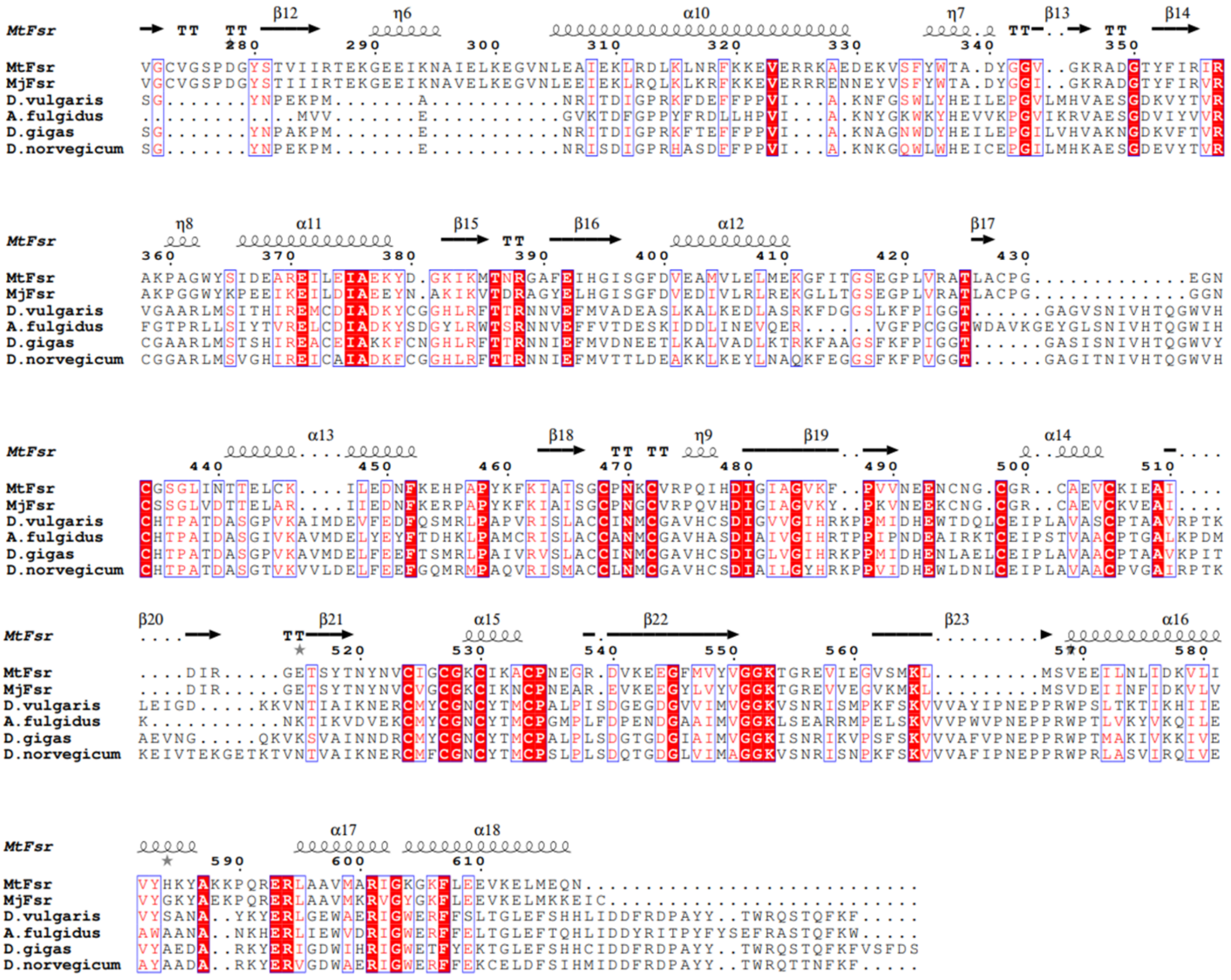

Supplementary Fig. 10. Sequence conservation across the C-terminal half of Fsr (*Mt*Fsr: 271-618) and DsrB. Perfectly conserved residues are highlighted with a red background. MtFsr: *M. thermolithotrophicus*; MjFsr: *M. jannaschii*. D.vulgaris: *Desulfovibrio vulgaris* (PDB 2V4J); A.fulgidus: *Archaeoglobus fulgidus* (PDB 3MM5); D.gigas: *Desulfovibrio gigas* (PDB 3OR1); D.norvegicum: *Desulfomicrobium norvegicum* (PDB 2XSJ). Sequence alignment was done using Clustal Omega[16], secondary structure representation was performed with ESPript 3.0[13]. Arg355 seems not conserved in *A. fulgidus*, *D. vulgaris* and *D. norvegicum* due to a shift of two residues.

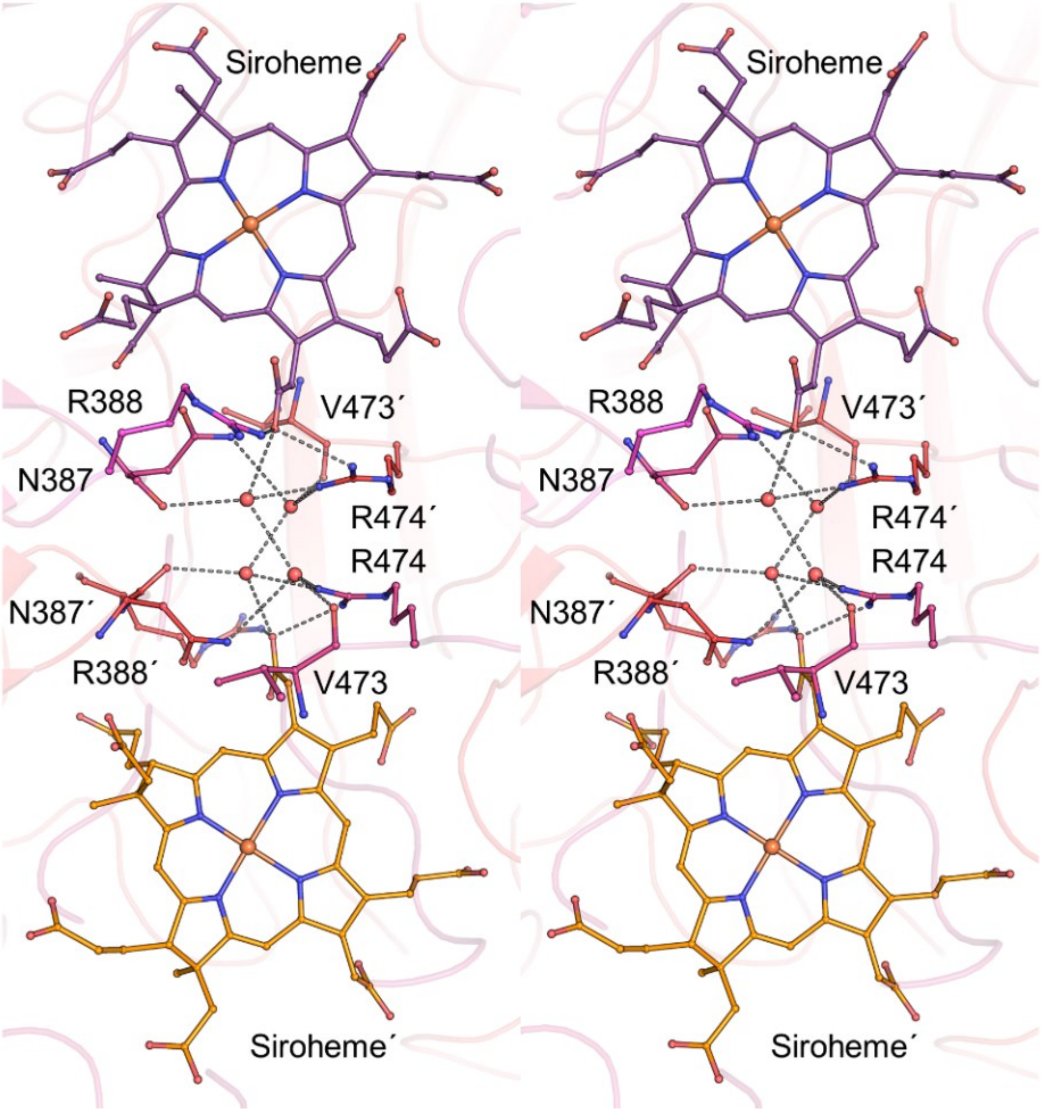

Supplementary Fig. 11. Stereo view of the intra-dimeric sirohemes in *Mt*Fsr, in which each chain is differently coloured. Primed labels indicate residues belonging to the dimeric partner. Sirohemes, water and residues involved in the channel are represented as balls and sticks. The distance between the two closest siroheme carboxylate groups is 9.4 Å. This close contact would theoretically allow an internal electron transfer between both sirohemes.

Supplementary Table 1. Chemical list of reagents used in this article.

Compound	Company	Catalog number
Gas mixture N_2/H_2, 95:5	Air Liquide	ARCAL F5
Potassium ferricyanide	Sigma Aldrich	244023
Tris ultrapure	AppliChem	A1086
Methylene blue hydrate	Sigma Aldrich	66720
Resorufin	Sigma Aldrich	73144
Indigo carmine	Sigma Aldrich	131164
2-Hydroxy-1,4-naphthochinon	Sigma Aldrich	H46805
Sodium anthraquinone-2-sulfonate	Sigma Aldrich	123242
Phenosafranin	Sigma Aldrich	199648
Safranin T	Sigma Aldrich	S8884
Neutral red	Sigma Aldrich	N4638
Benzylviologen	Sigma Aldrich	271845
Methylviologen dichloride hydrate	Sigma Aldrich	856177
PD10 desalting (Sephadex GH-25)	Cytiva	17085101
Sodium sulfite	Sigma Aldrich	S4672
Copper (II) sulfate pentahydrate	Sigma Aldrich	209198
Disodium EDTA	AppliChem	131669
KCl	Roth	6781.1
NaCl	Roth	3957.1
$NaHCO_3$	Roth	6885.1
$CaCl_2 \cdot 2\ H_2O$	Roth/Merck	5239.1/1.02382.
$MgCl_2 \cdot 6\ H_2O$	Roth	2189.1
NH_4Cl	Merck	1,011,451,000
Nitrilotriacetic acid	Sigma Aldrich	72560
$FeCl_2 \cdot 4\ H_2O$	Sigma Aldrich	380024
$Na_2SeO_3 \cdot 5\ H_2O$	Merck	1.06607
$Na_2WO_4 \cdot 2\ H_2O$	Merck	1,066,730,250
$Na_2MoO_4 \cdot 2\ H_2O$	Merck	1065210100
Resazurin Sodium Salt	Sigma	R7017
PIPES	Roth	9156.4
Sodium hydroxide pellets	Applichem	AP131687-1211
Tris Hydrochlorid	Roth	9090.3
MES	Roth	4259.4
$MnCl_2 \cdot 4\ H_2O$	Roth	T881.3
$FeCl_3 \cdot 6\ H_2O$	Fluka	44944
$CaCl_2 \cdot 2\ H_2O$	Roth/Merck	5239.1/1.02382.
$CoCl_2 \cdot 6\ H_2O$	Roth	T889.2

$ZnCl_2$	Merck	1,088,160,250
$NiCl_2 \cdot 6\ H_2O$	Sigma Aldrich	654507
VCl_3	Sigma Aldrich	208272
Sodium sulfite	Sigma Aldrich	71988
Sodium sulfate	Sigma Aldrich	1.06649
Sodium sulfide	Sigma Aldrich	407410
1.4-dithiothreitol	Neolab/BioFroxx	1111GR100
Bradford (Bio-Rad-Protein Assay)	Thermo Fisher	23246
Tris Hydrochlorid	Roth	9090.3
Ammoniumsulfate	Merck Applichem	1.01211
Glycerol	Applichem	141339.1211
di-Potassium hydrogen phosphate	Roth	P749.2
Potassium dihydrogen phosphate	Roth	3904.1
Ammoniumhydrogencarbonat	Roth	T871.1
Tris	Serva	37181.02
Sodium borohydride	Sigma Aldrich	452882
HCl, 25%	Sigma Aldrich	100316
Tricine	Roth	6977.3
Bis-Tris	Roth	9140
Sodium deoxycholate	Fluka	30970
Dodecyl maltoside	Roth	CN26.2
Trypsin from bovine pancreas	Sigma Aldrich	T8003
Sodium nitrite	Sigma Aldrich	563218

References

1 Johnson, E. F. & Mukhopadhyay, B. A new type of sulfite reductase, a novel coenzyme F_{420}-dependent enzyme, from the methanarchaeon *Methanocaldococcus jannaschii*. *J Biol Chem* **280**, 38776-38786, doi:10.1074/jbc.M503492200 (2005).

2 Vitt, S. *et al.* The F_{420}-reducing [NiFe]-hydrogenase complex from *Methanothermobacter marburgensis*, the first X-ray structure of a Group 3 family member. *Journal of Molecular Biology* **426**, 2813-2826, doi:10.1016/j.jmb.2014.05.024 (2014).

3 Brüggemann, H., Falinski, F. & Deppenmeier, U. Structure of the $F_{420}H_2$:quinone oxidoreductase of *Archaeoglobus fulgidus* identification and overproduction of the $F_{420}H_2$-oxidizing subunit. *Eur J Biochem* **267**, 5810-5814, doi:10.1046/j.1432-1327.2000.01657.x (2000).

4 Bäumer, S. *et al.* The $F_{420}H_2$:heterodisulfide oxidoreductase system from *Methanosarcina* species. 2-Hydroxyphenazine mediates electron transfer from $F_{420}H_2$ dehydrogenase to heterodisulfide reductase. *Febs Lett* **428**, 295-298, doi:10.1016/s0014-5793(98)00555-9 (1998).

5 Susanti, D. & Mukhopadhyay, B. An intertwined evolutionary history of methanogenic archaea and sulfate reduction. *Plos One* **7**, doi:ARTN e4531310.1371/journal.pone.0045313 (2012).

6 Raaijmakers, H. *et al.* Gene Sequence and the 1.8 Å Crystal Structure of the Tungsten-Containing Formate Dehydrogenase from *Desulfovibrio gigas*. *Structure* **10**, 1261-1272, doi:https://doi.org/10.1016/S0969-2126(02)00826-2 (2002).

7 Yu, H. *et al.* Comparative genomics and proteomic analysis of assimilatory sulfate reduction pathways in anaerobic methanotrophic archaea. *Front Microbiol* **9**, 2917, doi:10.3389/fmicb.2018.02917 (2018).

8 Crane, B. R. & Getzoff, E. D. The relationship between structure and function for the sulfite reductases. *Curr Opin Struc Biol* **6**, 744-756, doi:Doi 10.1016/S0959-440x(96)80003-0 (1996).

9 Dhillon, A., Goswami, S., Riley, M., Teske, A. & Sogin, M. Domain evolution and functional diversification of sulfite reductases. *Astrobiology* **5**, 18-29, doi:10.1089/ast.2005.5.18 (2005).

10 Kim, J. Y., Nakayama, M., Toyota, H., Kurisu, G. & Hase, T. Structural and mutational studies of an electron transfer complex of maize sulfite reductase and ferredoxin. *J Biochem* **160**, 101-109, doi:10.1093/jb/mvw016 (2016).

11 Yoshimoto, A. & Sato, R. Studies on yeast sulfite reductase. I. Purification and characterization. *Biochim Biophys Acta* **153**, 555-575, doi:10.1016/0005-2728(68)90185-0 (1968).

12 Sievers, F. *et al.* Fast, scalable generation of high-quality protein multiple sequence alignments using Clustal Omega. *Mol Syst Biol* **7**, 539, doi:10.1038/msb.2011.75 (2011).

13 Robert, X. & Gouet, P. Deciphering key features in protein structures with the new ENDscript server. *Nucleic Acids Res* **42**, W320-324, doi:10.1093/nar/gku316 (2014).

14 Edgar, R. C. MUSCLE: a multiple sequence alignment method with reduced time and space complexity. *BMC Bioinformatics* **5**, 113, doi:10.1186/1471-2105-5-113 (2004).
15 Tamura, K., Stecher, G. & Kumar, S. MEGA11: Molecular evolutionary genetics analysis version 11. *Mol Biol Evol* **38**, 3022-3027, doi:10.1093/molbev/msab120 (2021).
16 Sievers, F. *et al.* Fast, scalable generation of high-quality protein multiple sequence alignments using Clustal Omega. *Molecular Systems Biology* **7**, doi:ARTN 53910.1038/msb.2011.75 (2011).
