# Supplementary material for: Structures of the sulfite detoxifying F420-dependent enzyme from Methanococcales
Source: Nat Chem Biol. 2023 Jan 19;19(6):695–702. doi: 10.1038/s41589-022-01232-y (PMC10229431; doi:10.1038/s41589-022-01232-y)
Supplement: Supplementary file 2 — Reporting Summary [file 41589_2022_1232_MOESM2_ESM.pdf]

nature portfolio

Corresponding author(s): Wagner Tristan

Last updated by author(s): Nov 18, 2022

Reporting Summary

Nature Portfolio wishes to improve the reproducibility of the work that we publish. This form provides structure for consistency and transparency in reporting. For further information on Nature Portfolio policies, see our Editorial Policies and the Editorial Policy Checklist.

Statistics

For all statistical analyses, confirm that the following items are present in the figure legend, table legend, main text, or Methods section.

n/a	Confirmed	
☐	☒	The exact sample size (n) for each experimental group/condition, given as a discrete number and unit of measurement
☐	☒	A statement on whether measurements were taken from distinct samples or whether the same sample was measured repeatedly
☒	☐	The statistical test(s) used AND whether they are one- or two-sided<br>*Only common tests should be described solely by name; describe more complex techniques in the Methods section.*
☒	☐	A description of all covariates tested
☒	☐	A description of any assumptions or corrections, such as tests of normality and adjustment for multiple comparisons
☐	☒	A full description of the statistical parameters including central tendency (e.g. means) or other basic estimates (e.g. regression coefficient) AND variation (e.g. standard deviation) or associated estimates of uncertainty (e.g. confidence intervals)
☒	☐	For null hypothesis testing, the test statistic (e.g. F, t, r) with confidence intervals, effect sizes, degrees of freedom and P value noted<br>*Give P values as exact values whenever suitable.*
☒	☐	For Bayesian analysis, information on the choice of priors and Markov chain Monte Carlo settings
☒	☐	For hierarchical and complex designs, identification of the appropriate level for tests and full reporting of outcomes
☒	☐	Estimates of effect sizes (e.g. Cohen's d, Pearson's r), indicating how they were calculated

Our web collection on statistics for biologists contains articles on many of the points above.

Software and code

Policy information about availability of computer code

Data collection	X-ray Data collection were performed at European synchrotrons and are all mentioned and acknowledged in the manuscript. A spectrophotometer UV/VIS UVmini-1240 SHIMADZU was used for growth measurement. A pH meter from Mettler Toledo, Seven Excellence S400 Basic, was used to measure redox titration. All EPR spectra were recorded on a Bruker Elexsys E580 X band spectrometer (digitally upgraded) with a 4122HQE cavity linked to an ESR 900 Oxford Instruments helium flow cryostat. Cryocooling was performed by a Stinger (Cold Edge Technologies) closed-cycle cryostat driven by an F-70 Sumitomo helium compressor. UV/visible spectra were recorded on an Agilent Cary 60 UV-Vis. Kinetic parameters were measured on a FLUOstar Omega multi-mode Microplate Reader.
Data analysis	All software used for this study have been mentioned and cited appropriately in our manuscript. autoPROC version 1.5.5, CRANK-2 version 2.0.227, PHENIX version 1.19.2-4158 for MjFsr and v_1.20.1-4487 for MtFsr, COOT version 0.8.9.2, Open-Source PYMOL version 2.2.0. EPR spectra were simulated with Easyspin (version 5.2.33, running under MATLAB 2021b.). Superpose (LSQKAB from CCP4i package) version 7.0.078. Excel version 16 (16.0.5356.1000).

For manuscripts utilizing custom algorithms or software that are central to the research but not yet described in published literature, software must be made available to editors and reviewers. We strongly encourage code deposition in a community repository (e.g. GitHub). See the Nature Portfolio guidelines for submitting code & software for further information.

Data

Policy information about availability of data

All manuscripts must include a data availability statement. This statement should provide the following information, where applicable:

- Accession codes, unique identifiers, or web links for publicly available datasets
- A description of any restrictions on data availability
- For clinical datasets or third party data, please ensure that the statement adheres to our policy

Structure factors and pdb files have been deposited to the Protein Data Bank and are fully available under the following codes: 7NP8 for MjFsr and 7NPA for MtFsr. X-ray crystallography raw data have been deposited on Zenodo (doi: 10.5281/zenodo.4751126). All data are available.

Human research participants

Policy information about studies involving human research participants and Sex and Gender in Research.

Reporting on sex and gender	Not applicable
Population characteristics	Not applicable
Recruitment	Not applicable
Ethics oversight	Not applicable

Note that full information on the approval of the study protocol must also be provided in the manuscript.

Field-specific reporting

Please select the one below that is the best fit for your research. If you are not sure, read the appropriate sections before making your selection.

☒ Life sciences ☐ Behavioural & social sciences ☐ Ecological, evolutionary & environmental sciences

For a reference copy of the document with all sections, see nature.com/documents/nr-reporting-summary-flat.pdf

Life sciences study design

All studies must disclose on these points even when the disclosure is negative.

Sample size	Three biological replicates were performed for the growth experiments. For all enzymatic assays, experimental triplicates were performed. Sample sizes were chosen based on statistical relevancy. All other sample sizes used to acquire experimental data are mentioned in the material and method section and Extended Data Table.
Data exclusions	No data was excluded from the analyses.
Replication	Replicates are stated in the legends and methods. All attempts were successfully replicated.
Randomization	Randomization were applied to generate the Rfree set
Blinding	N/A. Each biological and biochemical experiment in this study is rationally designed and leads to a specific conclusion.

Reporting for specific materials, systems and methods

We require information from authors about some types of materials, experimental systems and methods used in many studies. Here, indicate whether each material, system or method listed is relevant to your study. If you are not sure if a list item applies to your research, read the appropriate section before selecting a response.

Materials & experimental systems

n/a	Involved in the study	
☒	☐	Antibodies
☒	☐	Eukaryotic cell lines
☒	☐	Palaeontology and archaeology
☒	☐	Animals and other organisms
☒	☐	Clinical data
☒	☐	Dual use research of concern

Methods

n/a	Involved in the study	
☒	☐	ChIP-seq
☒	☐	Flow cytometry
☒	☐	MRI-based neuroimaging
