# Supplementary material for: Structures of the sulfite detoxifying F420-dependent enzyme from Methanococcales
Source: Nat Chem Biol. 2023 Jan 19;19(6):695–702. doi: 10.1038/s41589-022-01232-y (PMC10229431; doi:10.1038/s41589-022-01232-y)

Uncropped scans of gels from Extended Data Fig. 2

Extended Data Fig. 2b

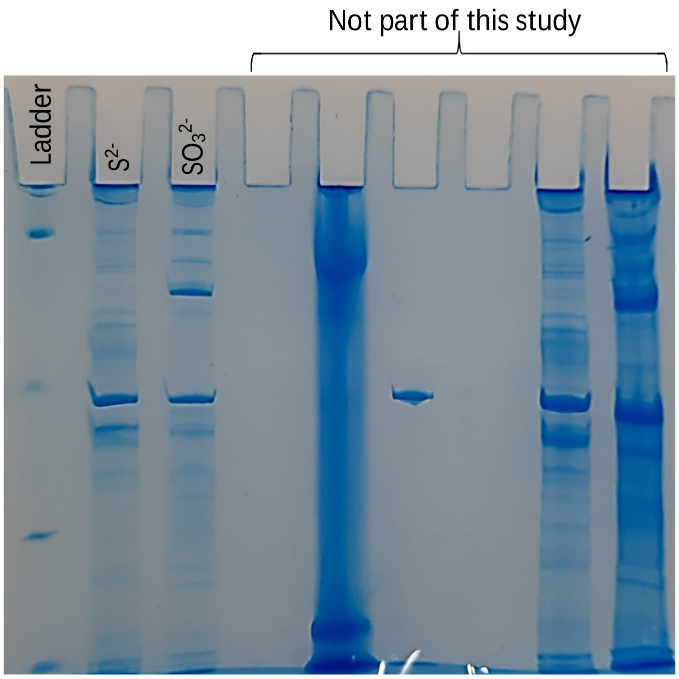

Extended Data Fig. 2c

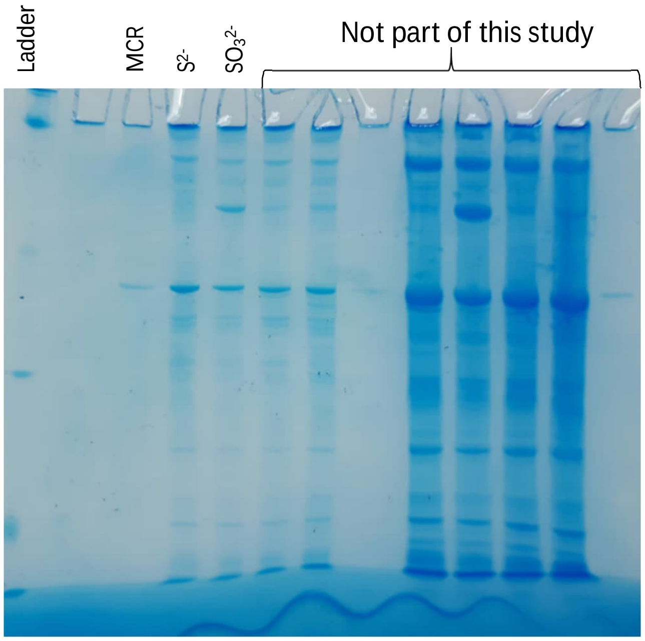

Extended Data Fig. 2d

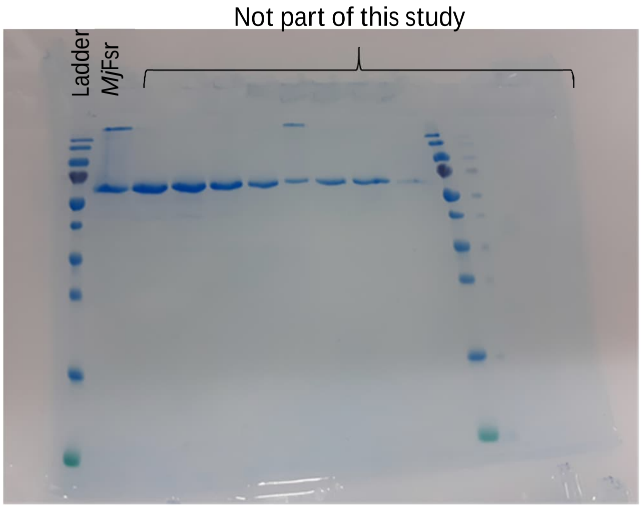

Extended Data Fig. 2e

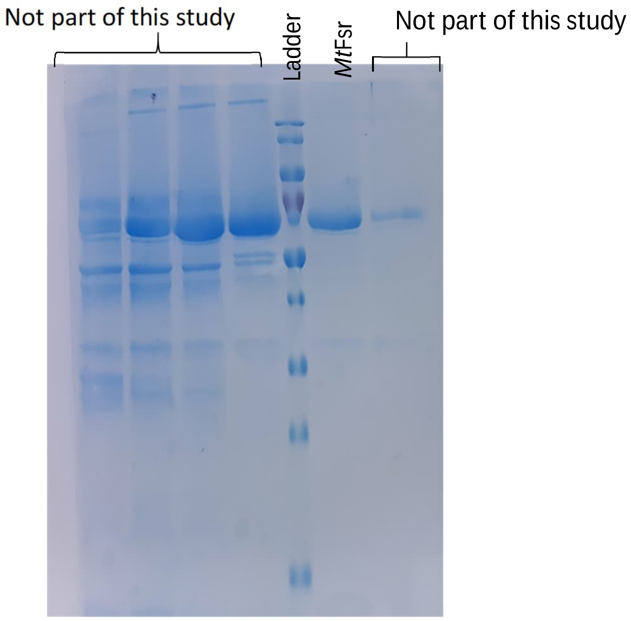

Supplement: Supplementary file 5 — Statistical source data for Extended Data Fig. 2. [file 41589_2022_1232_MOESM5_ESM.pdf]
